# Transcriptomic and metabolomic profiling of drought-tolerant and susceptible sesame genotypes in response to drought stress

**DOI:** 10.1186/s12870-019-1880-1

**Published:** 2019-06-20

**Authors:** Jun You, Yujuan Zhang, Aili Liu, Donghua Li, Xiao Wang, Komivi Dossa, Rong Zhou, Jingyin Yu, Yanxin Zhang, Linhai Wang, Xiurong Zhang

**Affiliations:** 1 Key Laboratory of Biology and Genetic Improvement of Oil Crops of the Ministry of Agriculture and Rural Affairs, Oil Crops Research Institute of the Chinese Academy of Agricultural Sciences, Wuhan, 430062 China; 2 Special Economic Crop Research Center of Shandon Academy of Agricultural Sciences, Shandong Cotton Research Center, Jinan, 250100 China; 3 Quality Inspection and Test Center for Oilseed Products, Ministry of Agriculture and Rural Affairs, Wuhan, 430062 China; 4 Centre d’Etudes Régional pour l’Amélioration de l’Adaptation à la Sécheresse (CERAAS), Thiès, 3320 Sénégal

**Keywords:** Sesame, Drought stress, Transcriptome, Metabolome, Abscisic acid, Amino acids

## Abstract

**Background:**

Sesame is an important oil crop due to its high oil, antioxidant, and protein content. Drought stress is a major abiotic stress that affects sesame production as well as the quality of sesame seed. To reveal the adaptive mechanism of sesame in response to water deficient conditions, transcriptomic and metabolomics were applied in drought-tolerant (DT) and drought-susceptible (DS) sesame genotypes.

**Results:**

Transcriptomic analysis reveals a set of core drought-responsive genes (684 up-regulated and 1346 down-regulated) in sesame that was robustly differently expressed in both genotypes. Most enriched drought-responsive genes are mainly involved in protein processing in endoplasmic reticulum, plant hormone signal transduction photosynthesis, lipid metabolism, and amino acid metabolism. Drought-susceptible genotype was more disturbed by drought stress at both transcriptional and metabolic levels, since more drought-responsive genes/metabolites were identified in DS. Drought-responsive genes associated with stress response, amino acid metabolism, and reactive oxygen species scavenging were more enriched or activated in DT. According to the partial least-squares discriminate analysis, the most important metabolites which were accumulated under drought stress in both genotypes includes ABA, amino acids, and organic acids. Especially, higher levels of ABA, proline, arginine, lysine, aromatic and branched chain amino acids, GABA, saccharopine, 2-aminoadipate, and allantoin were found in DT under stress condition. Combination of transcriptomic and metabolomic analysis highlights the important role of amino acid metabolism (especially saccharopine pathway) and ABA metabolism and signaling pathway for drought tolerance in sesame.

**Conclusion:**

The results of the present study provide valuable information for better understanding the molecular mechanism underlying drought tolerance of sesame, and also provide useful clues for the genetic improvement of drought tolerance in sesame.

**Electronic supplementary material:**

The online version of this article (10.1186/s12870-019-1880-1) contains supplementary material, which is available to authorized users.

## Background

Sesame (*Sesamum indicum* L), an ancient oil crop, is a popular ingredient in cuisines across the world. Sesame seeds are well known for their high oil content (~ 58%) and protein content (~ 25%) [1]. Sesame oil is regarded as superior quality oil due to a high content of unsaturated-fatty acid (~ 85%) and the presence of antioxidants (such as sesamin, sesamolin and tocopherols) [2]. Sesame is traditionally grown in drought-prone and marginal areas with sub-optimal water and nutrients supply. Although sesame has adapted to semi-arid conditions, it is sensitive to drought stresses at vegetative and reproduction stages, leading to reduce growth and loss in yield [3–6]. In addition, the quality of sesame seed, such as oil and protein content, also reduced by severe water stress [7]. Therefore, it is essential to understand the complex mechanisms underlying drought resistance in sesame and apply acquired knowledge for developing drought-resistant sesame cultivars [8].

Drought stress, the most important limiting environmental factors for sustainable agriculture, is responsible for the largest loses of global food productivity [9, 10]. With the development of molecule biotechnology, the molecular mechanism of drought response in plants has been gradually unveiled through functional genomics approaches. External drought stimuli is perceived by unknown sensors on the membrane, and then the signals are delivered through multiple signaling pathways, resulting in the expression of drought-responsive genes so as to confer drought tolerance in the plants [11, 12]. A multitude of signaling molecules, such as intracellular Ca^2+^, abscisic acid (ABA), inositol phosphate, and reactive oxygen species (ROS), are important for drought signal transduction [13]. The ABA signaling pathway is central to drought stress responses in plants, and it has been clearly elucidated by the identification of ABA receptors and other core signaling components [14]. Stress induced ABA perception by the ABA receptor PYR/PYL/RCAR suppresses the phosphatase activities of Group A PP2Cs, results in the activation of SnRK2s, which further phosphorylation or activation of downstream targets such as bZIP transcription factors and ion channels [14–16]. Transcription factors (TFs) are important components involved in transcriptional regulatory network functioning in drought abiotic stress responses. Members of DREB or CBF, bZIP, MYB, MYC, and NAC families have been characterized with roles in the regulation of plant drought responses through ABA-dependent and/or ABA-independent manner [12, 17]. With the development of high-throughput sequencing technologies, RNA-seq is extensively used to unravel the molecular basis of drought responses in many plant species [18–23]. Transcriptome analysis of most studies revealed that changes occur in photosynthesis, hormone signal transduction, amino acid metabolism, carbohydrate metabolism, secondary metabolites, as well as fatty acid metabolism in response to drought stress [20, 21, 23].

An obvious example for metabolites involved in plant stress response is the accumulation of sugar (such as trehalose and fructan) or amino acids (such as proline) that serve as osmoprotectant under drought stress. Mass spectrometric based global metabolic profiling, which targets a broader spectrum of metabolites, provides a comprehensive platform for investigating the metabolic reprogramming of plants under environmental stress [24, 25]. Recently, metabolic analyses have confirmed that changes in primary and secondary metabolites are associated with drought responses in several plant species. In *Arabidopsis*, the levels of most amino acids (such as proline, glutamine, tryptophan, alanine, aspartate, ornithine, isoleucine, leucine, valine), intermediates from TCA cycle (such as 2-oxoglutarate, *cis*-aconitate, and succinate), flavonoids (such as quercetin and cyanidin) and lipids (such as glycosylinositolphosphoceramides and acylated steryl glycosides) were increased under drought stress [26–28]. Similar changes in the levels of metabolites including amino acids and organic acid under drought stress were also reported in crop plants, such as rice [20], maize [29], and barley [30].

High-throughput multi-omics technologies are proving useful to meet the challenges in various research disciplines of crop plants [31–33]. Since the molecular mechanisms of abiotic stress resistance in sesame are largely unknown, functional genomics approaches involving transcriptomic and metabolomic profiling of the plant subjected to environmental stress including drought are required in sesame. Transcriptomic experiments have been carried out in sesame to study the response to drought and waterlogging stress in root [34, 35]. However, there are no studies to date reporting the analysis of drought response in leaves of sesame by integrating transcriptomics and metabolomics. In this study, we characterized transcriptional and metabolic profiling in two sesame genotypes with contrasting ability to cope with drought stress, and identified candidate genes and key metabolic pathways involved in drought response in sesame by integrated transcriptomic and metabolomic analysis.

## Results

### Variation of two sesame genotypes response to drought stress

**Fig. 1:**
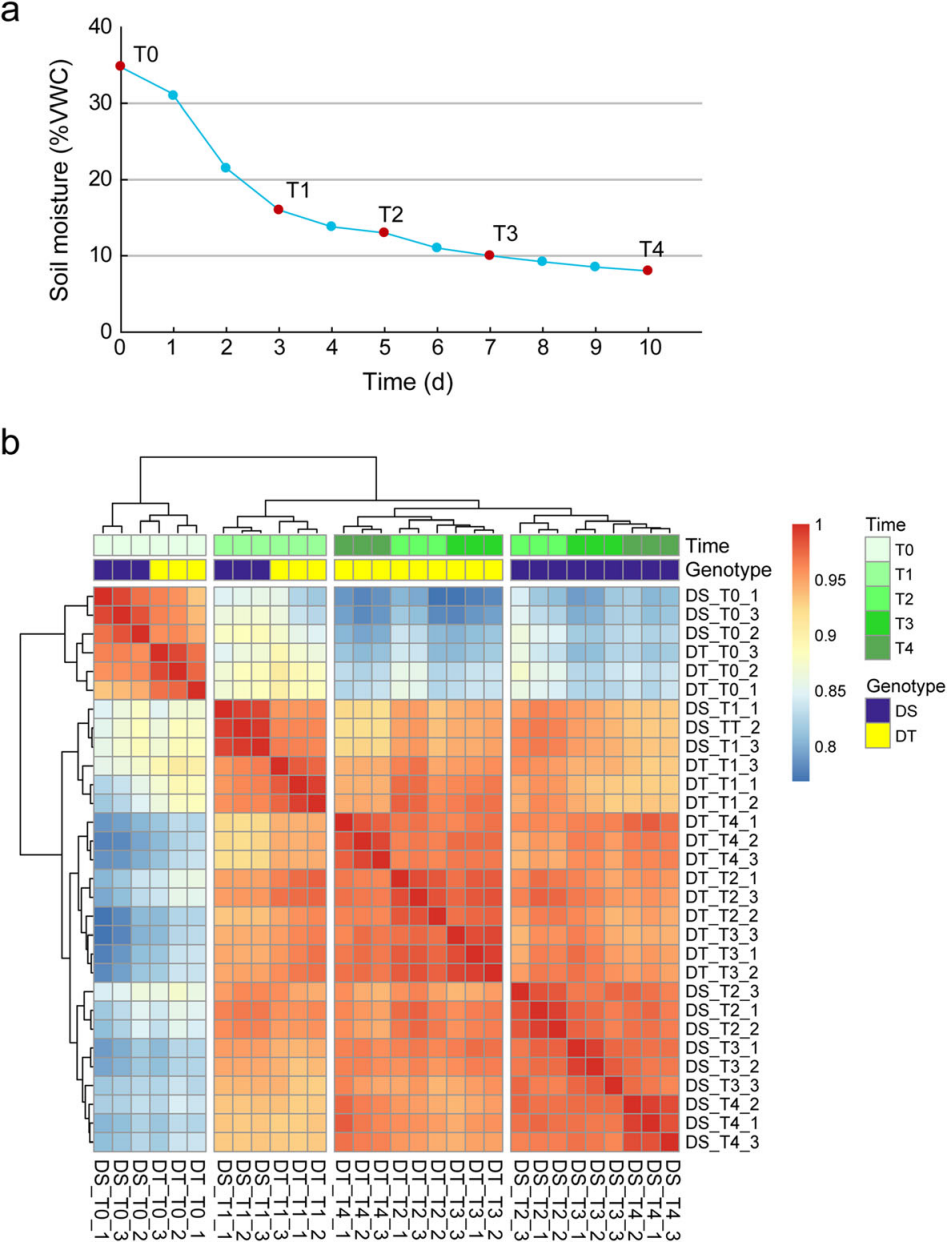
Samples collection and samples correlation of all the samples. **a** Changes of soil moisture during the drought stress. T0, T1, T2, T3 and T4 indicated plants stressed for 0, 3, 5, 7 and 10 days, respectively, were collected. **b** Samples correlation between the 30 samples**.** The heatmap represents Spearman’s rank correlation coefficients between pairs of samples based on global expression profiles in the drought-tolerant (DT) and drought-susceptible (DS) genotypes

Two sesame genotypes (ZZM3743 and ZZM3330) were submitted to drought stress, and significant differences in wilting and seedling survival were observed between the two genotypes. In the process of increasing water scarcity, the drought-susceptible genotype (ZZM3743, DS) showed wilting much earlier than the drought-tolerant genotype (ZZM3330, DT) (Additional file 1: Figure S1a). After drought stress for 15 days, plants were subjected to a rewatering treatment. After recovery, we found that more than 60% of DT plants were recovered, whereas only 20% of the DS plants were recovered, significantly (*t* test, *P* < 0.01) lower than the DT (Additional file 1: Figure S1b). To determine whether ROS level is linked to the different tolerance of these two sesame genotypes, malondialdehyde (MDA), a biomarker of oxidative stress, was measured as described previously [34]. The results showed that the MDA level was increased in leaves subjected to drought stress in both genotypes, but its content significantly (*t* test, *P* < 0.01) higher in the DS compared to DT under drought stress conditions (Additional file 1: Figure S1c). To reveal the drought tolerance mechanism of sesame, leaf tissue of two sesame genotypes were collected at five time points (T0, T1, T2, T3, and T4) with different stress intensity (monitored by soil moisture) during the drought stress treatment for transcription or metabolic analysis (Fig. 1a).

### Transcriptional characteristics of sesame response to drought stress

Total mRNA from leaf samples were sequenced using the Illumina paired-end sequencing method with three biological replicates per time point. The resulting set of 30 samples yielded more than 626 million clean reads. Over 81.66% of the reads were mapped to the sesame genome (Additional file 2: Table S1). A total of 20,226 and 19,848 unigenes were obtained from DS and DT sesame genotypes with FPKM values > 0.1 at least one sample, respectively (Additional file 1: Figure S2a). The expression of 17,932 and 17,839 genes was detected at all time points in DS and DT, respectively. Over 96% of the genes (19,597 genes) were detected in both DS and DT genotypes (Additional file 1: Figure S2b). To investigate the reproducibility of biological replicates and the relationship of different samples (genotype/treatment/time point), a correlation analysis was performed based on the global gene expression pattern. The high reproducibility between the biological replicates was illustrated through spearman correlation analysis. As shown in Fig. 1b, all the 30 samples were classed into four main groups. The control sample (T0) of DT and DS was clearly divergent from all the samples under drought stress treatment. Interestingly, samples of the two genotypes from early stress stages (T1) were clustered together. Whereas, when the stress becomes more severe (T2 to T4), the samples from the same genotype were clustered together, supporting the great difference of drought tolerance between DT and DS.

**Fig. 2:**
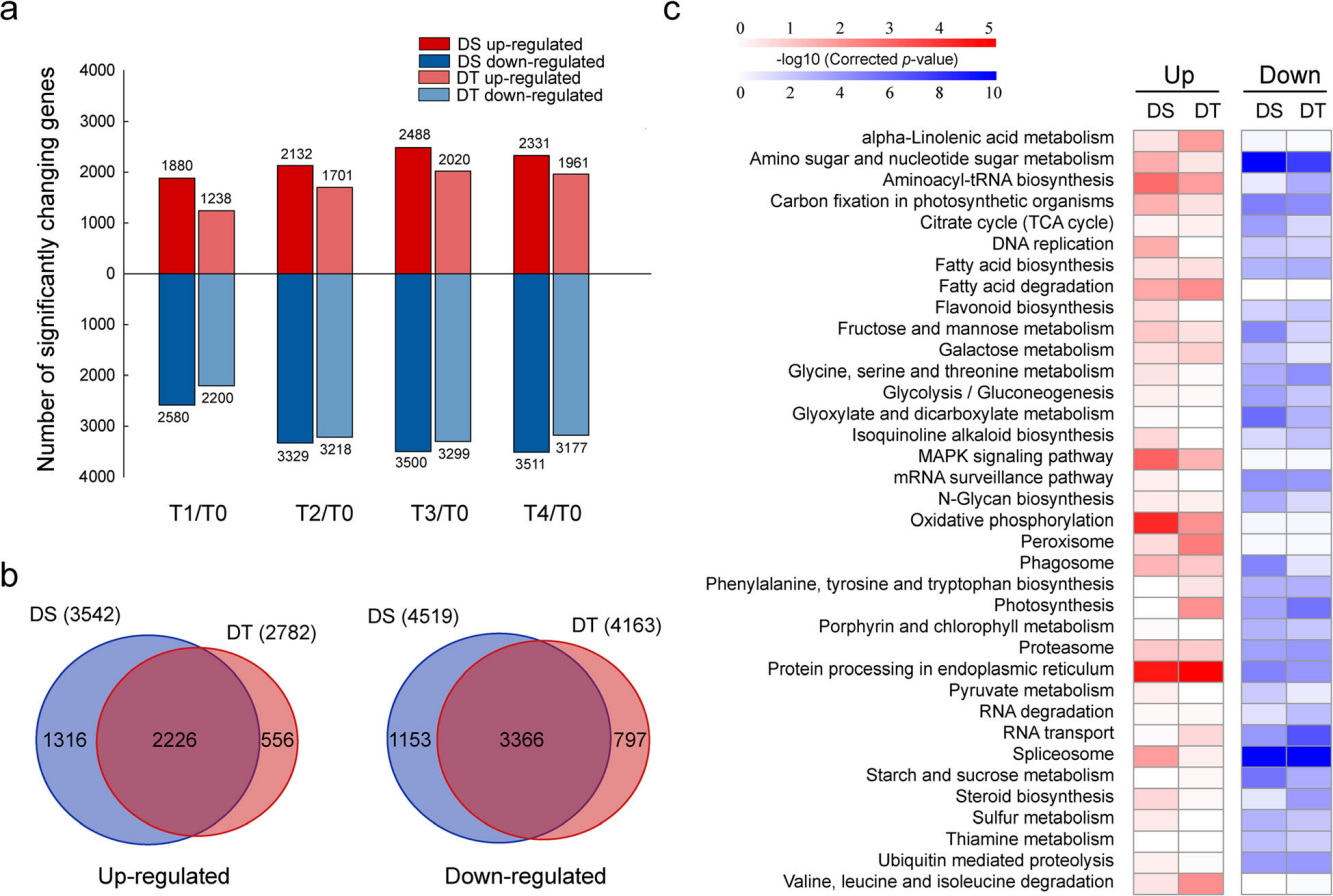
Comparison of up-regulated and down-regulated genes in drought-tolerant (DT) and drought-susceptible (DS) genotypes. **a** Number of up- and down-regulated differentially expressed genes identified in DT and DS response to drought stress. **b** Overlap among genes significantly up- and down-regulated by drought stress in DT and DS. **c** KEGG Pathway impacted by drought stress in DT and DS. KEGG pathway enrichment analysis was performed for lists of significantly up- and down-regulated genes for each genotype. The heatmap presents statistical significance (log10-transformed corrected *p* value) of KEGG pathway term over-representation

To investigate drought-responsive genes in sesame, the differentially expressed genes (DEGs) were identified at the criteria of false discovery rate (FDR) < 0.01 and |log2FC (fold change)| > 1. As shown in Fig. 2a, DT exhibited slightly less DEGs (ranging from 3438 to 5319) than DS (ranging from 4460 to 5988) during the drought treatment periods. Additionally, the number of down-regulated genes is higher than that of up-regulated genes at each time point in both genotypes. We identified 874 and 1046 genes up-regulated; 1786 and 1920 genes down-regulated in all the four time points during drought stress in DT and DS, respectively (Additional file 1: Figure S3a). The overlapped genes between genotypes were further compared to identify the core genes involved in sesame response to drought stress. As shown in Additional file 1: Figure S3b, a total of 684 up-regulated genes and 1346 down-regulated genes were overlapped between the two genotypes (Additional file 3: Table S2). To validate the RNA-seq data, the gene expression changes of twelve randomly selected DEGs were analyzed by real-time quantitative RT-PCR (qRT-PCR). Although the fold changes of selected genes were different between RNA-seq and qRT-PCR, the trends of changes were the same (Additional file 1: Figure S4).

GO and KEGG pathway enrichment analysis was employed to explore the potential functions of the core drought-responsive genes in sesame. The 684 up-regulated core drought-responsive genes were enriched in response to stress (abiotic stimulus, temperature stimulus, oxygen-containing compound, water deprivation, and chemical), metabolism (organonitrogen compound biosynthetic process, macromolecule metabolic process, small molecule catabolic process, and lipid oxidation), protein folding and aging (Additional file 1: Figure S5a). Biological process GO terms enriched in down-regulated core genes were function related to photosynthesis, carbohydrate metabolic process, lipid metabolic process, cell wall organization or biogenesis, and meristem growth (Additional file 1: Figure S5b). KEGG pathway enrichment analysis revealed that genes involved in protein processing in endoplasmic reticulum, galactose metabolism, and plant hormone signal transduction were up-regulated by drought stress. Pathways of photosynthesis, fatty acid biosynthesis, sugar and amino acid metabolism (amino sugar and nucleotide sugar metabolism, phenylalanine, tyrosine and tryptophan biosynthesis, glycine, serine and threonine metabolism), DNA replication and ribosome were enriched in down-regulated core genes (Additional file 1: Figure S6).

### Different drought-responsive genes between DT and DS

We further evaluated the difference of global gene expression in each genotype in response to drought stress by comparing the transcriptome change under drought treatments between genotypes. A total of 2782 genes were up-regulated and 4163 genes were down-regulated by drought stress in DT at least one time point. Likewise, 3542 up-regulated and 4519 down-regulated genes in DS were detected at least one time point during drought stress. Of these DEGs, 2226 drought-induced genes and 3366 drought-repressed genes overlapped between the two genotypes (Fig. 2b).

The enrichment of KEGG pathways and GO terms of DEGs was compared between DT and DS to reveal the different drought responsive of two genotypes (Fig. 2c and Additional file 1: Figure S7). Four pathways including alpha-Linolenic acid metabolism, valine, leucine and isoleucine degradation, photosynthesis and peroxisome were preferably enriched in up-regulated DEGs in DT. While, pathways of amino sugar and nucleotide sugar metabolism, DNA replication, and spliceosome were preferably enriched in up-regulated DEGs in DS. There were more shared KEGG pathways for down-regulated DEGs in the two genotypes, including spliceosome, amino sugar and nucleotide sugar metabolism, and ubiquitin mediated proteolysis. In comparison to the drought-tolerance genotype, pathways of citrate cycle (TCA cycle), fructose and mannose metabolism, and glyoxylate and dicarboxylate metabolism and phagosome were enriched in down-regulated DEGs in DS. Similar to the results of KEGG enrichment, many enriched GO terms were shared between DS and DT. However, genes associated with stress response were preferably enriched in up-regulated DEGs in DT. These results indicate that the drought-tolerance genotype has both common pathways and unique pathways to cope with drought stress.

### Differential expressed genes between DT and DS

**Fig. 3:**
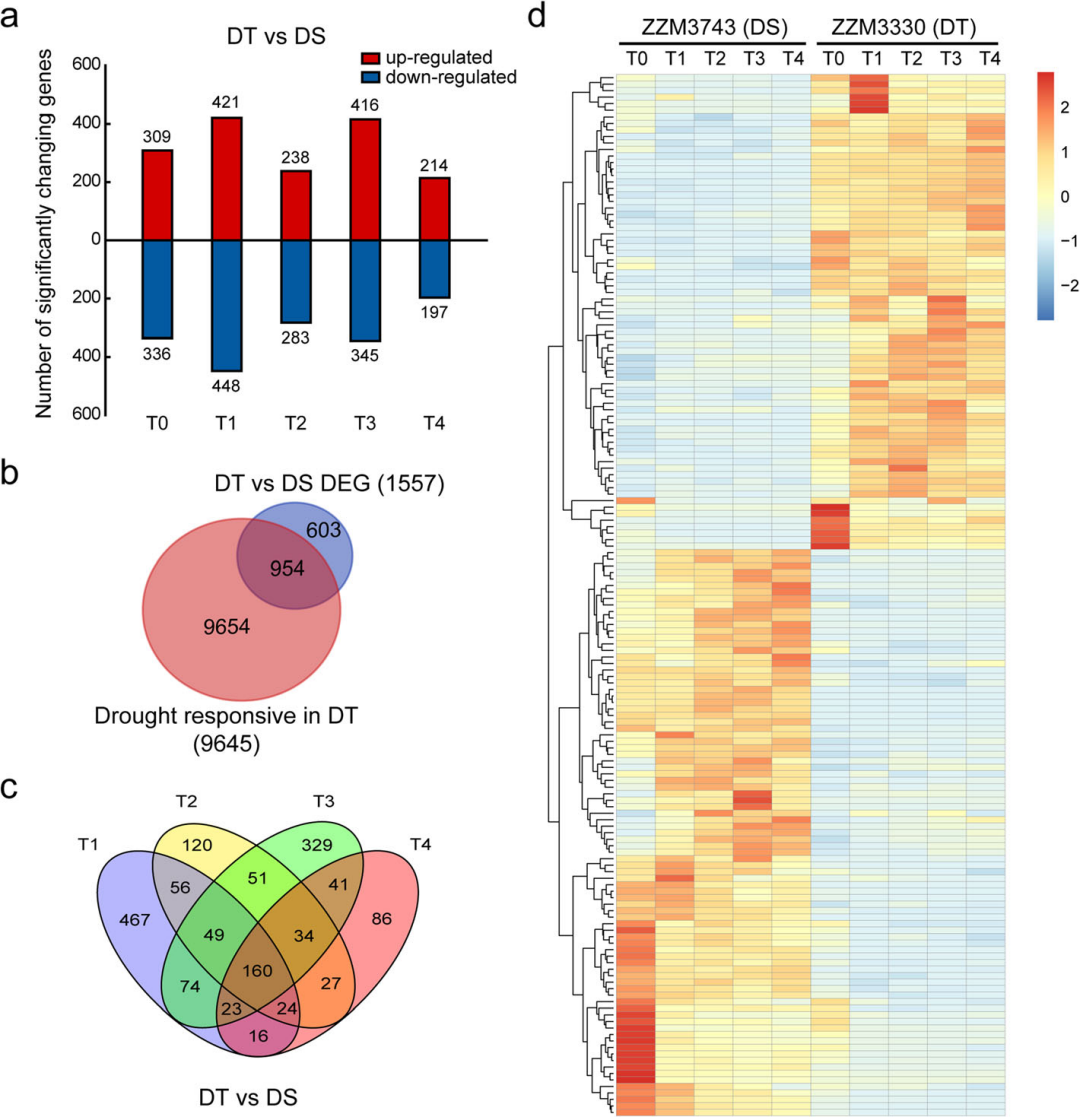
Differential expression genes between drought-tolerant (DT) and drought-susceptible (DS) genotypes. **a** Number of up- and down-regulated differentially expressed genes (DEGs) between DT and DS. **b** Overlap among drought-responsive genes and DEGs between two genotypes. **c** Overlap of DEGs between two genotypes under drought stress (T1, T2, T3, and T4). **d** Expression patterns of the 160 common DEGs between DT and DS under drought conditions. The heatmap presents normalized FPKM expression values

Genes conferring higher drought resistance in sesame were investigated by comparing DEGs between DT and DS. At a threshold of a 2-fold change in expression level, 1871 genes were significantly (FDR < 0.01) and differentially expressed between DT and DS at least one time point. Of these DEGs, 948 were up-regulated and 923 were down-regulated in DT compared to the levels in DS (Additional file 1: Figure S8), indicating that the gene expression profiles were remarkably distinct during the drought stress of these two contrasting sesame genotypes. Under normal growth condition (T0), 645 genes were differentially expressed between DT and DS (Fig. 3a). GO analysis revealed that these genes were enriched in biological process related to terpenoid and isoprenoid metabolic process (Additional file 1: Figure S9a). A total of 1557 genes were differentially expressed during the drought stress period (T1-T4) between DT and DS. Among these genes, 954 genes were response to drought stress in DT (Fig. 3b), suggesting that these genes may function in drought tolerance in DT. Based on Gene ontology analysis, it was indicated that the majority of genes differentially expressed between DT and DS during the drought stress were enriched in biological process related to macromolecule metabolic process, nitrogen compound metabolic process, terpene biosynthetic process, protein modification process and organelle organization (Additional file 1: Figure S9b). KEGG pathway enrichment analysis revealed that these genes were enriched in pathway related to phenylpropanoid biosynthesis, cyanoamino acid metabolism, glutathione metabolism, stilbenoid, diarylheptanoid and gingerol biosynthesis, and alpha-Linolenic acid metabolism (Additional file 1: Figure S9d). Based on the overlap analysis, we identified 160 genes showing differential expression between DT and DS in all the four time points of drought stress (Fig. 3c, Additional file 4: Table S3). Hierarchical clustering analysis further classified these genes into 2 major groups based on their expression pattern (Fig. 3d). More than half of these genes exhibit differential expression between DT and DS even under normal growth conditions. Most of these DEGs were cytochrome P450, DnaJ homolog protein, ABC transporter and UDP-glycosyltransferase. Of the 160 DEGs, genes encoding for ABA 8′-hydroxylase, arginase, and glutathione S-transferase were already known for their stress association in plants.

### Metabolic characteristics of sesame response to drought stress

**Fig. 4:**
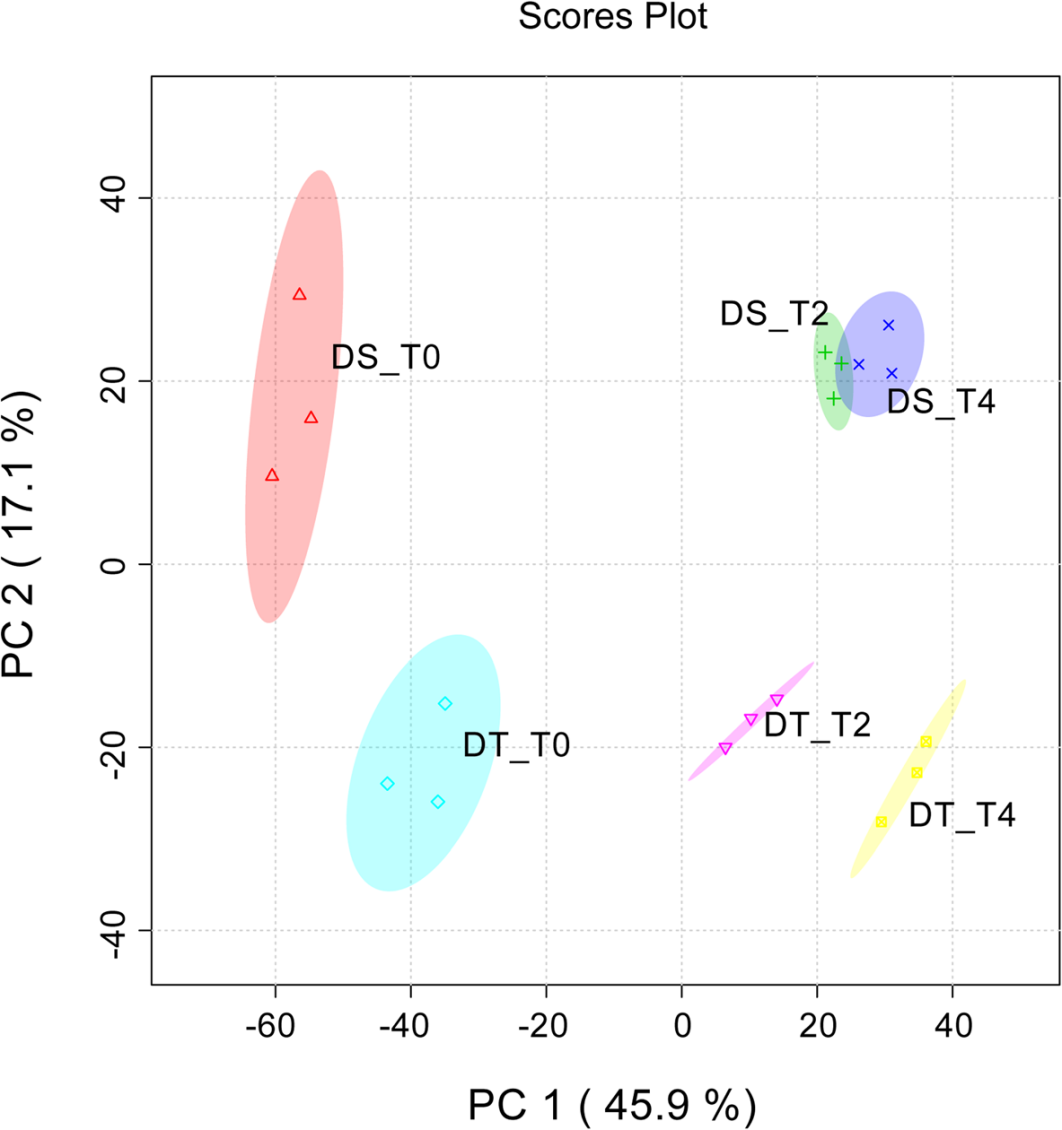
Principal component analysis (PCA) of metabolic profiles of DT and DS under control and drought stress

To investigate the metabolic response to drought stress in sesame, plant samples collected at T0, T2 and T4 were analyzed from each genotype using global untargeted metabolite analysis based on ultra performance liquid chromatography/mass spectrometry (UPLC/MS) and gas chromatography/mass spectrometry (GC/MS) platform. The combined platforms detected a total of 345 unique named metabolites, of which 285 compounds could be identified in all samples from two genotypes (Additional file 1: Figure S10). Different types of compounds were detected in GC/MS and UPLC/MS platforms including: amino acids; organic acids; carbohydrates; nucleosides, nucleotides, and their analogs; alkaloids and their derivatives, among others. Principal component analysis (PCA) was performed to reduce the dimensionality of the data and visualize the relationship among samples. The first principal component (PC1) explained 45.9% of total variation, while the second principal component (PC2) explained 17.1% variation across the data set (Fig. 4). The scores plot between the PC1 and PC2 shows clear separation by PC2 between the different genotypes. The time of exposure of the plants to stress conditions is well separated by PC1. This indicates changes in the metabolite profiles caused by the drought stress as well as by the differences between two cultivars in control samples and in response to drought.

**Fig. 5:**
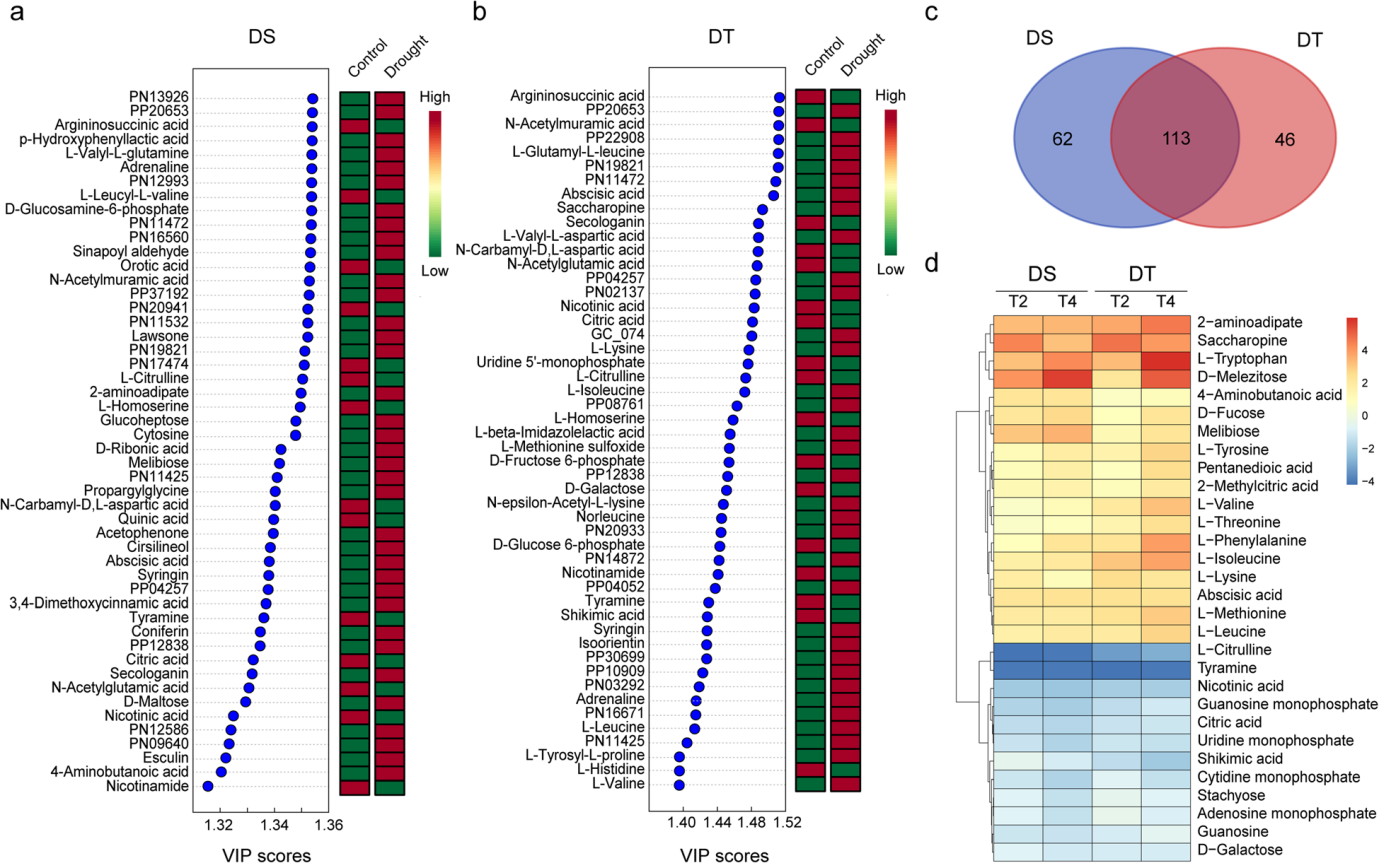
Important metabolites in response to drought stress identified by partial least squares-discriminant analysis (PLS-DA) in DT and DS. Fifty top metabolites according to the VIP (variable importance in projection) score in DS (**a**) and DT (**b**) are shown. Metabolites with complex name were indicated by Peak_ID presented in Additional files 5 and 6. Colored boxes indicate the relative concentrations of the corresponding metabolite in each group. **c** Overlap among drought-responsive metabolites in DT and DS. **d** Heatmap illustrating the Log2 fold change of selected common drought-responsive metabolites in the leaves of two sesame gonetypes underdrought conditions at two time points

To identify the drought-responsive metabolites in sesame, the PLS-DA (partial least squares-discriminant analysis) was performed for drought and control conditions on DT and DS varieties separately. Drought stress treatment explained 47.0 and 55.1% of total variation in DT and DS, respectively (Additional file 1: Figure S11). On the basis of the VIP (variable importance in projection) score > 1, 159 and 174 drought-responsive metabolites with important variations were identified in DT and DS, respectively (Additional file 5: Table S4 and Additional file 6: Table S5). The top 50 drought-responsive metabolites in DT and DS and their changing patterns were presented in Fig. 5a and b. By intersection analysis, 113 drought-responsive metabolites across the different genotypes were identified (Fig. 5c). Most of these robust drought-responsive metabolites were consistently increased or decreased in DT and DS varieties. Metabolites which were highly accumulated under drought stress including phytohormone ABA, amino acids (e.g., tryptophan, phenylalanine, valine, leucine, tyrosine, saccharopine and 2-aminoadipate), 4-aminobutanoic acid (GABA), and organic acids (e.g., glutaric acid, and 2-methylcitric acid). Conversely, some nucleosides and nucleotides (e.g., guanosine, uridine, adenosine monophosphate, cytidine monophosphate, guanosine monophosphate, and uridine monophosphate), and sugar (D-galactose and stachyose) were reduced under drought stress (Fig. 5d). Furthermore, 62 and 46 metabolites were found associated with drought stress specifically in DT and DS, respectively. For instance, the well-known stress responsive osmolyte, proline, was identified as drought-responsive metabolites specific in DT with a VIP of 1.09.

### Differences in metabolite content between DT and DS

To investigate the difference of two varieties in drought resistance at the metabolic level, the important metabolites with different contents between DT and DS were identified through PLS-DA (Additional file 1: Figure S12). In total of 108 metabolites showed a significant difference between the two genotypes with VIP score > 1 (Additional file 7: Table S6). Some metabolites, including D-Saccharic acid, 4-hydroxyproline, Cyclic GMP, adenine, and guanine, had a higher content in DT than in DS under all conditions, whereas the ononin and luteolin 7-glucoside had a reduced content. Many of metabolites represented different patterns between two genotypes under drought stress condition. For example, lactobionic acid, benzoic acid, putrescine and many amino acids were highly accumulated in DT compared to DS under drought stress condition, whereas arbutin and tetronic acid were highly accumulated in DS. Especially, some drought-induced amino acids, such as asparagine, arginine, tyrosine and lysine, had higher content in DT under stress condition.

### Drought effect on amino acid metabolism

Analysis of the relevant pathways affected by water-deficit conditions based on drought-responsive metabolites was performed by the Pathway Analysis of Metaboanalyst4 using *Arabidopsis thaliana* pathway library. Totally, 119 drought-responsive metabolites were mapped to different steps of fifty pathways (Additional file 8: Table S7), of which two amino acid pathways (Arginine and proline metabolism, Alanine, aspartate and glutamate metabolism) were significantly disturbed by drought stress (FDR < 0.05; pathway impact values > 0.2).

**Fig. 6:**
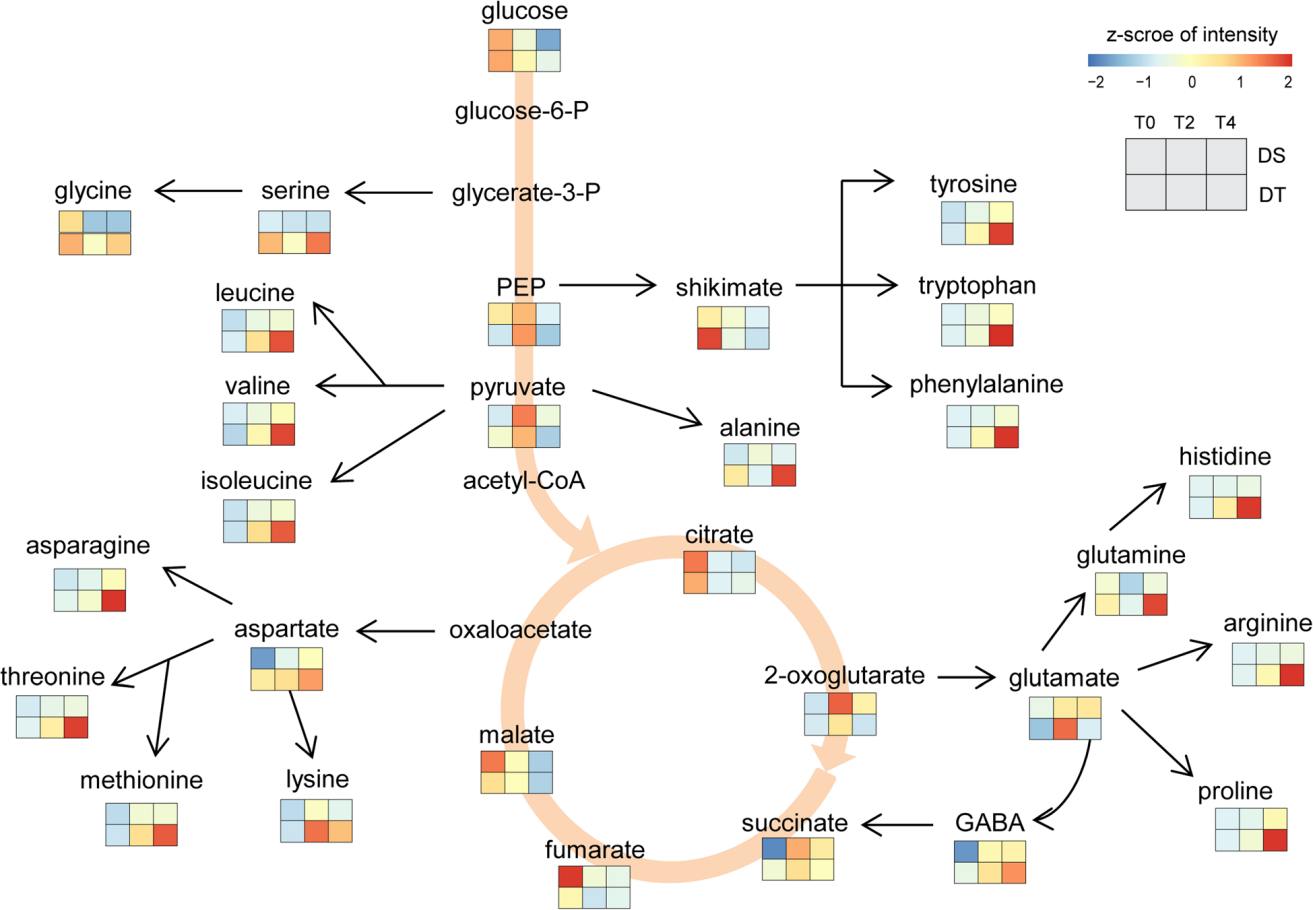
Metabolic changes involved in amino acid pathways and tricarboxylic acid cycle under drought stress in DT and DS. Each graph represents the normalized intensity of corresponding metabolite in three sampling points from two genotypes

Free amino acid levels for tryptophan, phenylalanine, isoleucine, valine, tyrosine, leucine, lysine, saccharopine were all significantly increased (log2FC > 1, FDR < 0.01) under drought stress treatment in both DT and DS. Whereas, the significantly accumulation of histidine, arginine, methionine, proline, and threonine under drought stress was only observed in DT (Fig. 6). Most of these drought-induced amino acids including tryptophan, arginine, leucine, isoleucine proline, histidine, and saccharopine, were more abundant in DT under drought stress (especially at T4). Corresponding to the accumulation of amino acids under drought stress, the expression of some amino acid biosynthesis related genes was up-regulated. Branched chain aminotransferase catalyzes the last step of BCAA (branched-chain amino acids) biosynthesis. Expression of the *LOC105162194*, a member of branched chain aminotransferase family, was induced by drought in both genotypes. GABA, a non-protein amino acid derived from the decarboxylation of glutamate, increased significantly (FDR < 0.05) in DS. But the relative levels of GABA were greater in DT for both normal and drought stress conditions (Fig. 6). The transcript level of *LOC105168543*, which encodes the GABA biosynthetic enzyme glutamate decarboxylase, increased under water-deficit conditions in both genotypes.

**Fig. 7:**
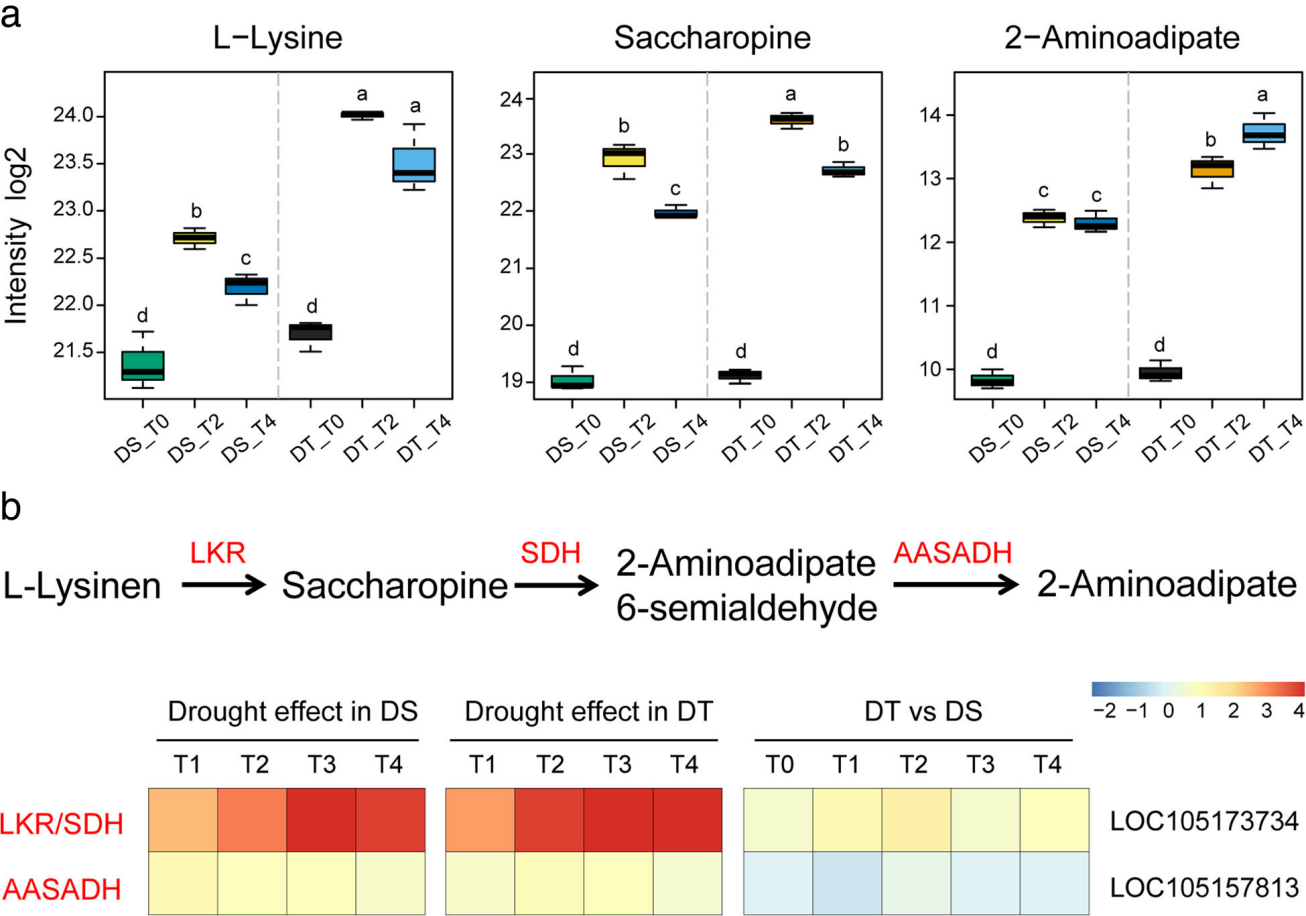
Modulation of saccharopine pathway under drought stress in DT and DS. **a** Differences in the metabolites involved in saccharopine pathway in DT and DS. Different letters indicate significant differences between groups (*P* < 0.05, turkey’s test). **b** The relative expression of genes involved in saccharopine pathway in DT and DS. Data are presented as heatmaps of a log2 transformed fold change

It is worth noting that saccharopine and 2-aminoadipate, two intermediates of lysine catabolism (also called saccharopine pathway), accumulated in both genotypes under drought stress (Fig.7a). Correspondingly, the relative intensity of lysine increased at T2, then fell back at T4 during the drought stress. However, all these metabolites have higher abundance in the tolerant variety. Lysine degradation through the saccharopine pathway consists of three enzymatic steps that are catalysed by lysine-ketoglutarate reductase (LKR), saccharopine dehydrogenase (SDH) and aminoadipic semialdehyde dehydrogenase (AASADH). The first two steps of this pathway were catalyzed by a bifunctional LKR/SDH enzyme encoded by a single gene. As showed in Fig.7b, sesame LKR/SDH gene (*LOC105173734*) was up-regulated under drought stress in both DS and DT, and more abundance of transcript was observed in DT. The transcript level of *LOC105157813*, encodes AASADH, was also increased under water-deficit conditions in both genotypes.

### Expression of genes involved ABA metabolism

**Fig. 8:**
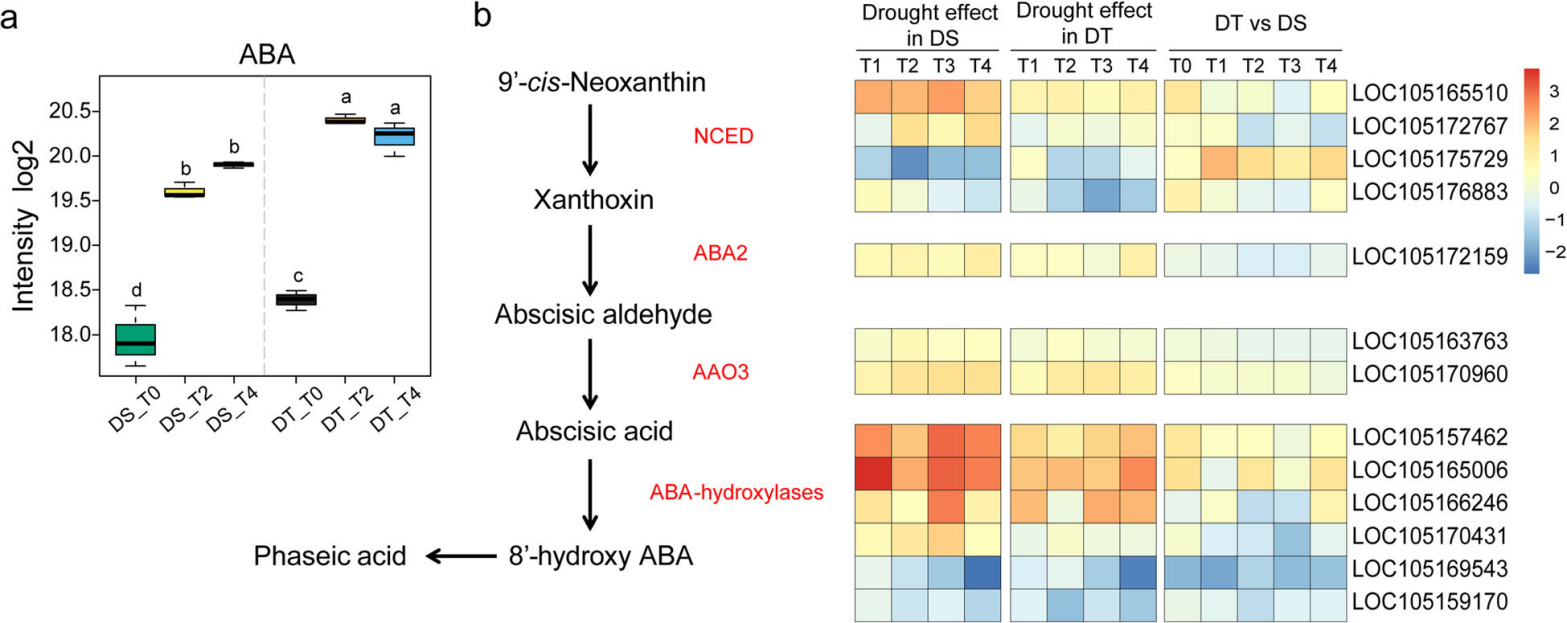
Modulation of ABA metabolism pathway under drought stress in DT and DS. **a** ABA levels in DT and DS. Different letters indicate significant differences between groups (*P* < 0.05, turkey’s test). **b** The relative expression of the genes involved in ABA metabolism pathway in DT and DS. Data are presented as heatmaps of a log2 transformed fold change

As mentioned above, we observed the accumulation of ABA in the leaves of drought stressed plants in both genotypes. Moreover, the relative level of ABA was consistently higher in DT (Fig. 8a). Then, the expression pattern of genes involved in ABA metabolism pathway under drought stress was investigated in two genotypes (Fig. 8b). The 9-*cis*-epoxycarotenoid dioxygenase (NCED) is thought to be a key enzyme in ABA biosynthesis. Four genes encoding NCED in sesame showed expression change under drought stress, and most of these NCED showed higher expression level in DT than in DS under control and drought stress condition. Hydroxylation of ABA is the key step in ABA catabolism, which is catalyzed by a group of cytochrome P450 enzymes known as ABA-hydroxylases. Six genes annotated to be ABA-hydroxylases in sesame, and all of them responded differently to drought stress. The expression of two ABA-hydroxylases genes, *LOC105169543* and *LOC105159170*, decreased under drought treatment for all genotypes. Whereas, other four genes up-regulated under drought stress, most notably in DS. Comparison of two genotypes, these ABA-hydroxylases genes showed higher abundance in DS than in DT in most time points.

## Discussion

Sesame is an important oil crop due to its high oil, antioxidant, and protein content. Adverse environmental stresses occurring at the vegetative and/or reproduction stage not only decrease the yield in sesame, but also affect the quality of sesame seed [4, 7]. Therefore, it is important to identify major stress tolerance mechanisms in sesame to help formulate strategies for genetic improvement of abiotic stress resistance. To this end, we investigated the transcriptomic and metabolic reprogramming in leaves of two sesame genotypes with contrasting drought tolerance exposed to gradual water deficit treatment. From a general view, a greater number of genes were either up or down-regulated, and more metabolites were changed in DS than DT in response to drought stress, suggesting the susceptible genotype was more disturbed by drought stress at both transcriptional and metabolic levels. The hyper-responsive to abiotic stress at the molecular level in susceptible genotype was also reported in other plant species [20, 36, 37]. This may indicate absence of homeostasis mechanisms in susceptible genotypes to mitigate the impact of water deficit.

Based on the overlapped DEGs, a set of core drought-responsive genes (2030 genes) that were differential expressed in both sesame genotypes was identified (Additional file 3: Table S2). Main group of up-regulated core genes encoding molecular chaperones, including heat shock protein (Hsp) 70, Hsp 90, Hsp100, small heat shock protein (sHsp) and Dna J proteins, which are responsible for protein folding, assembly, translocation and degradation in a broad array of cellular processes [38–40]. Genes commonly up-regulated by drought in both DT and DS were also represented by those encoding aquaporins and late embryogenesis abundant (LEA) proteins including dehydrins. These proteins are tightly correlated with acquisition of drought tolerance and accumulated in response to water deficit in various plant species [41, 42]. Among commonly down-regulated genes in all time points and genotypes, ribosomal protein genes were represented. It has been reported that ribosomal protein genes were regulated by various environmental conditions, which cause changes in ribosome compositions [43, 44]. Our data revealed that over 150 ribosomal protein genes were significantly down-regulated in responses to drought stress, indicating the protein translation of sesame may be affected by unfavorable conditions. In previously study, root transcriptomic profiles of sesame under drought stress were analyzed [34]. Comparative analysis of core drought-responsive gene sets in sesame leaves and roots released that the enriched GO terms are different between the two tissues (Additional file 3: Figure S13). Leaf-specific genes showed unique enriched GO terms related to photosynthesis, glucose 6-phosphate metabolic process, fatty acid biosynthetic and metabolic process, while enriched GO terms such as peroxidase activity, pectin catabolic process, ion binging and transport were found only in root tissue. These results suggested that there are tissue-specific adaption mechanisms in different tissues to cope with drought stress.

Reactive oxygen species (ROS) are significantly accumulated under abiotic stress conditions, which cause oxidative damage in cell [45]. Genes encoding ROS-scavenging enzymes, such as superoxide dismutase (SOD), catalase (CAT), and ascorbate peroxidase (APX) which are known to be important in ROS homeostasis and drought resistance [46, 47], were significantly up-regulated under drought stress in the current study. Glutaredoxins (GRXs) are small oxidoreductases that participate in defense against oxidative stress by either direct ROS scavenging or redox regulation of target proteins [48]. More GRXs were found up-regulated in DT genotype under drought condition indicating that they act positively to relieve cellular oxidative damage caused by drought in sesame as demonstrated in many other plants [49, 50]. In addition, some of the up-regulated genes were mapped on the pathways related to antioxidant metabolites such as glutathione metabolism, phenylpropanoid biosynthesis, and carotenoid biosynthesis. Glutathione S-transferase (GST), which involved in glutathione metabolism, plays vital roles in maintaining cellular redox homeostasis and mediating plant abiotic stress resistance [51]. Here, higher expression level of several drought-induce *GST* genes was found in DT genotype under drought condition, indicating that the expression of *GST* genes was closely associated with drought resistance in sesame.

The phytohormones are vital regulators in plant response to environmental stress by integrating external stimuli with complex regulatory networks. ABA is thought to be a key hormone in plant adaptation to drought conditions. Accumulation of cellular ABA under stress triggers the activation of ABA-responsive genes and the closure of stomata to reduce water loss [52]. ABA signaling pathway can be considered important to the drought resistance of sesame since higher levels of ABA were observed in DT genotype under both normal and stress conditions in the present study. Consistent with this, *LOC105169543* encoding ABA-hydroxylases, the enzyme responsible for ABA catabolism has significantly lower expression in DT than DS. In addition, genes encoding NCED, which is involved in ABA biosynthesis, showed higher expression level in DT than in DS. The accumulation of ABA activated the ABA-dependent transcription pathway in sesame plants exposed to drought stress. Several orthologs of *ABFs* (*ABA-Responsive-Element binding factors*) and *DREB2s* (*Dehydration-Responsive-Element-Binding Proteins 2*) which are involved in ABA-dependent pathway were up-regulated in responses to drought stress in both genotypes. We also found up-regulation of sesame homolog encoding NAC TF AtRD26 which functions as a transcriptional activator in the ABA signaling pathway [53]. In addition, the second most highly induced TF gene (*LOC105160292*) in DT encodes a protein similar to ATHB7 which plays a role in drought stress through ABA-dependent signal transduction pathway [54].

The increased of specific amino acid levels considered essential for plant stress tolerance by acting as osmolytes, precursors for energy-associated metabolites, ROS scavengers, as well potential regulatory and signaling molecules [55, 56]. The results presented in this study demonstrated an accumulation of large amounts of amino acids in sesame under drought stress, which was consistent with previous reports in other plant species [20, 26, 29]. Moreover, the relative intensity of some amino acids, including tryptophan, leucine, isoleucine, arginine, proline, histidine, saccharopine were higher in DT under stress condition, suggesting the metabolic pathways involving these amino acids were related to drought tolerance in sesame. In accordance with the metabolic profiles, transcriptomic analysis found that many genes involved in amino acid metabolism were modulated under drought stress.

Proline is known to accumulate under stress and considered to act as compatible osmolyte and radical scavenger [57]. Here, the significantly increase of proline was specifically found in stressed leaves of DT at T4 (7.19-fold change, FDR < 0.01). BCAAs (leucine, isoleucine and valine) and aromatic amino acids (tryptophan, phenylalanine and tyrosine) are also highly increased in DT during drought stresses in our study. It has been demonstrated that catabolism of BCAAs function as an alternative electron donor in the respiratory chain under stress condition [58]. BCAAs also play an important role in plant drought tolerance as an alternative source of respiratory substrates [26]. Aromatic amino acids are known as precursors of many natural products including, alkaloids, phytoalexins, auxins, and cell wall components, that play essential roles in plant growth and environmental responses [59]. In the present study, tryptophan is the most increased amino acids during drought stress in both DT and DS. Tryptophan is thought to play an important role in osmotic adjustment, stomatal regulation, and ROS scavenging under stress condition [60]. In addition, the accumulation of saccharopine and 2-aminoadipate highlight the activation of saccharopine pathway under drought stress in sesame. The saccharopine pathway is the major route for lysine catabolism. It has been reported that the saccharopine pathway genes, such as LKR/SDH and AASADH, were up-regulated in plant submitted to osmotic and /or salt stress. We also found the up-regulated of these enzyme genes in sesame drought response. Moulin et al. [61] reported that the induction of LKR/SDH in osmotically-stressed tissues is correlated with the increased accumulation of pipecolic acid, which acts as an osmoprotectant. Most importantly, over expression of soybean AASADH results in increased tolerance to salt and drought stress in transgenic plants [62]. All these results demonstrate the important role of saccharopine pathway in stress response in plants.

The pool size of the TCA cycle intermediates, such as citricate, malate and fumarate was materially reduced under drought stress, indicating that both the TCA cycle and glycolysis were inhibited by water deficiency. Accumulation of soluble sugar is reported in many plant species to protect plants against adverse growing conditions. Our metabolic profiling showed significantly accumulation of melezitose and fucose in sesame. Melezitose is reported to accumulate upon stress and associated with desiccation tolerance in plants [63]. Although the expression of raffinose family oligosaccharides biosynthesis genes is up-regulated under drought condition, the significantly accumulation of raffinose was not observed in the present study. 3′,5′-cyclic adenosine monophosphate (cAMP) and 3′,5′-Cyclic guanosine monophosphate (cGMP) are well known second messengers involved in many processes in plants including growth, differentiation, photosynthesis, and defense [64]. Here, cAMP and cGMP were found specially accumulated in DT under drought stress, and DT had a higher content of cAMP and cGMP than DS in both normal and stress conditions. As cAMP can induce proteins related to abiotic stress response [65], higher cAMP in DT may activate defense responses to help plants cope with adverse environment. We also recorded increased levels of allantoin, the intermediary metabolite of purine catabolism, at later stress stage (T4) in DT, but not in DS. It has been reported that allantoin accumulated in response to abiotic stresses and play important roles in mediating plant adaptation to stress. Nourimand and Todd [66] demonstrated that allantoin enhanced stress tolerance by activation of antioxidant mechanisms. In addition, *Arabidopsis* mutant that over-accumulation of allantoin showed enhanced abiotic stress tolerance by increasing ABA levels and activating stress-responsive gene expression [67].

## Conclusion

**Fig. 9:**
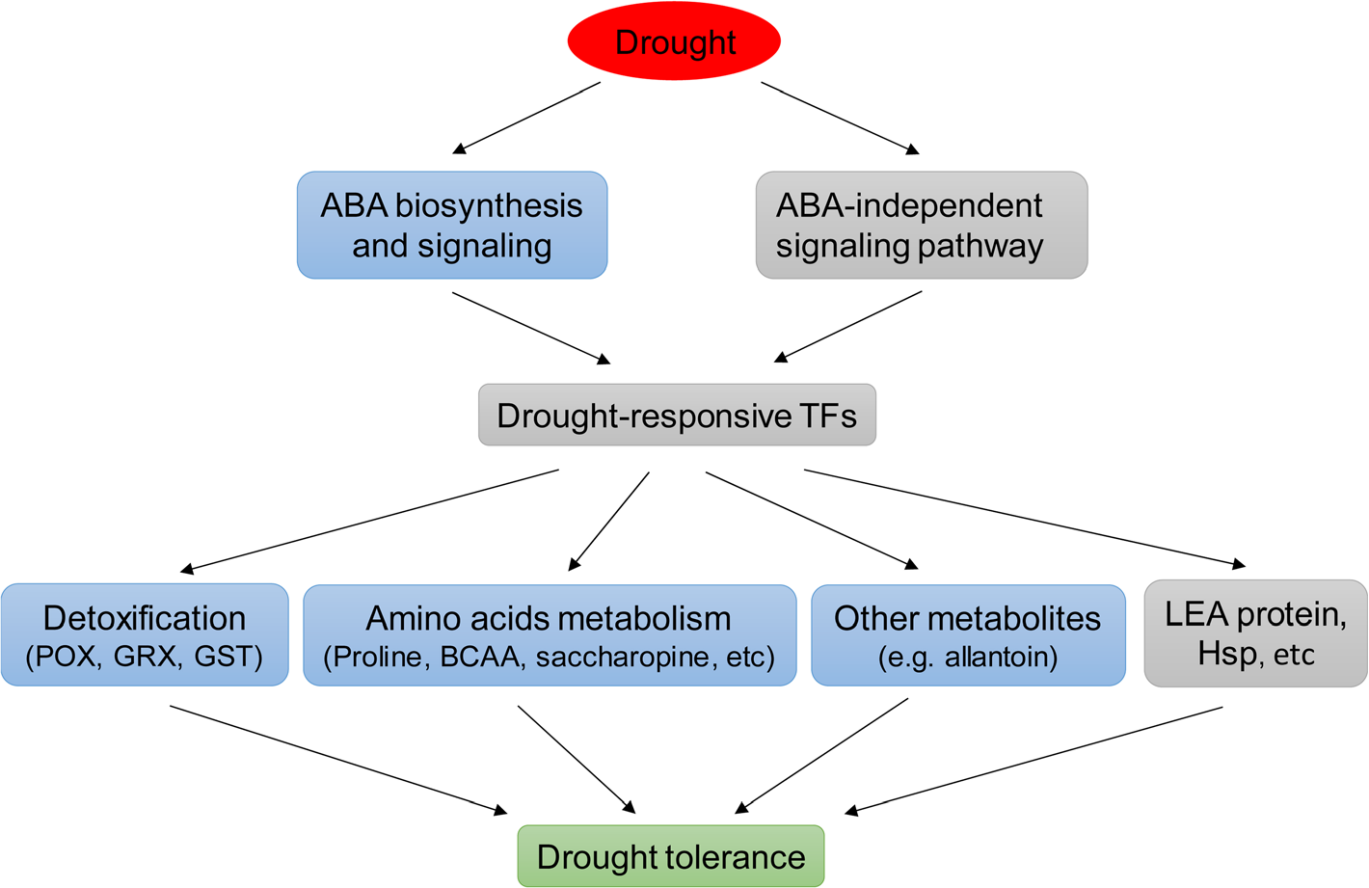
A model depicting drought tolerance of two sesame genotypes. Blue color boxes indicate elevation in expression of metabolic pathway genes or content of metabolites in DT compared to DS. ABA, abscisic acid; BCAA, branched-chain amino acids; GRX, Glutaredoxins; GST, Glutathione S-transferase; POX, Peroxidase

We comprehensively analyzed the global changes in transcript and metabolic profiles in two sesame genotypes with contrasting drought tolerance. Pathway analysis indicated up-regulation of genes involved in protein processing in endoplasmic reticulum, galactose metabolism and plant hormone signal transduction and down-regulation of photosynthesis in both genotypes under drought. We have shown that the drought-tolerant genotype was less affected by water deficit at both transcriptional and metabolic levels. Combined transcriptomic and metabolomic analysis suggested that drought tolerance of DT was found to be linked to (1) increased ABA levels and activated ABA signaling pathway, (2) enhanced amino acids metabolism and accumulated some stress-related amino acids, and (3) enhanced enzymatic activity or metabolic pathways associated with ROS-scavenging (Fig. 9). Functional validation of drought-responsive genes or metabolic pathways identified in this study will help to uncover the complexity of drought tolerance at the molecular level and will be useful for breeding drought-tolerant sesame cultivars.

## Materials and methods

### Plant material and stress treatment

The seeds of two sesame (*Sesamum indicum* L.) accessions, ZZM3743 (drought-susceptible, DS) and ZZM3330 (drought-tolerant, DT) were obtained from a large collection of sesame accessions preserved at the China National Gene Bank, Oil Crops Research Institute, Chinese Academy of Agricultural Sciences. Plants were sown in pots (25 cm diameter and 30 cm depth) containing 7 Kg of loam soil mixed with 10% of added compound fertilizer. The seedlings were thinned at 2 true leaves stage and 12 plants per pot were kept. The drought stress treatment was imposed at four-pair leaf stage (25 days after sowing). Soil moisture (VWC) of each pot was monitored using a Moisture Meter Takeme (China) over the entire experiment. Leaf samples from ten randomly selected plants were collected (as one biological replicate) when the soil moisture reached 16% (T1), 13% (T2), 10% (T3) and 8% (T4) during the drought stress treatments, as well as before stress (T0), respectively. A schematic sketch of the experiment is presented in Fig. 1. All the samples were harvested between 9:00 to 9:30 am to eliminate the potential influences by circadian or other environmental factors. For each time point, three replicates from each genotype were used for RNA extraction. A further three replicates of leaf samples collected at T0, T2 and T4 were used for metabolite analysis.

### RNA sequencing and genome mapping

Total RNA was isolated using the EASYspin Plus kit (Aidlab, China) according to the manufacturer’s instructions. The mRNA was enriched and purified with oligo (dT)-rich magnetic beads and then used to generate RNA-seq libraries with insert sizes ranging from 300 to 350 bp using TruSeq Stranded mRNA Sample Preparation Kit (Illumina). Finlay, the qualified libraries were sequenced using Illumina HiSeq™2500 with a 150 bp paired-end strategy in Seqhealth Technology Co., Ltd. (Wuhan, China). The quality of the raw data was assessed with FastQC. The adaptor sequences and low-quality sequence reads were removed from the raw data sets using Trimmomatic [68] with following parameters: ILLUMINACLIP:{truseq.adapter.fa}:2:30:5 LEADING:3 TRAILING:3 SLIDINGWINDOW:4:15 HEADCROP:0 MINLEN:36. After filtering, the clean reads were mapped to the reference genome sequence of sesame (https://www.ncbi.nlm.nih.gov/genome/11560) using Tophat2 [69].

### Quantification of gene expression and differential expression analysis

The expression levels of each gene were calculated and normalized by the corresponding Fragments Per Kilobase of transcript per Million mapped fragments (FPKM) with Cufflinks. The expression levels of twelve randomly selected genes were validated by qRT-PCR as described previously [70]. The list of primers is presented in Additional file 9: Table S8. Differential expression analysis of two conditions or groups was performed with the R-based package DESeq [71]. The resulting *P* values were adjusted using the Benjamini and Hochberg’s approach for controlling the false discovery rate. Genes with a false discovery rate (FDR) < 0.01 and a logarithm two-fold change |log2FC| ≥ 1 were defined as differentially expressed genes. Enrichment analyses of Gene Ontology (GO) and KEGG pathways based on the differentially expressed genes were performed by using the ClueGO plugin in Cytoscape [72]. A Benjamini-Hochberg corrected *p* value of < 0.05 based on two-sided hypergeometric tests was selected as the threshold for significant KEGG pathway or GO enrichment of the gene sets.

### Untargeted metabolomics analysis

Untargeted metabolomics profiling was performed using ultra performance liquid chromatography/mass spectrometry (UPLC/MS) and gas chromatography/mass spectrometry (GC/MS) platform (metaSysX GmbH, Potsdam-Golm, Germany). Sample preparation for metabolite extraction and measurement was performed as described by Giavalisco et al. [73]. UPLC/MS analysis was based on Waters ACQUITY Reversed Phase Ultra Performance Liquid Chromatography (RP-UPLC) coupled to a Thermo-Fisher Exactive mass spectrometer which consists of an ElectroSpray Ionization source (ESI) and an Orbitrap mass analyzer. Instrumental settings were previously described [73]. C18 columns were used for the hydrophilic measurements. Chromatograms were recorded in Full Scan MS mode (Mass Range [100–1500]). GC-MS data were obtained using an Agilent Technologies GC coupled to a Leco Pegasus HT mass spectrometer which consists of an EI ionization source and a TOF mass analyzer. The GC temperature programming began at 85 °C for 2 min, continued with 15 °C per min up to 360 °C.

### Metabolomics data processing and analysis

Extraction of the LC-MS data was accomplished with the software REFINER MS® 10.5 (GeneData, http://www.genedata.com). After extraction from the chromatograms, the data was processed, aligned and filtered using in-house software. The annotation of the content of the sample was accomplished by matching the extracted data from the chromatograms with internal library of reference compounds in terms of accurate mass and retention time. For GC-MS data processing and annotation, NetCDF files that were exported from Leco Pegasus software were imported to “R”. The Bioconductor package TargetSearch was used to transform retention time to retention index (RI), to align the chromatograms, to extract the peaks, and to annotate them by comparing the spectra and the RI to the Fiehn Library and to a user created library. Annotation of peaks was manually confirmed in Leco Pegasus. Analytes were quantified using a unique mass. Finally, the filtered data from all platforms was normalized to the weight of samples used for extraction and according to sample median intensity group-wise, and the resulting data matrices were used for further analysis.

Data matrices with intensity of metabolite features at three time points under both drought and control conditions for two varieties were uploaded to the MetaboAnalyst 4.0 server (http://www.metaboanalyst.ca) for univariate and multivariate statistical analyses. Univariate analysis (two-paired *t* test) was applied to calculate the statistical significance and fold change of the metabolites between two time points (drought over control). False discovery rate (FDR) was used for controlling multiple testing. The supervised multivariate method, PLS-DA (partial least squares-discriminant analysis), was used to maximize the metabolome difference between the control and drought treated samples, as well as the difference between two varieties. The relative importance of each metabolite to the PLS-DA model was checked using a parameter called the variable importance in projection (VIP). Metabolite with VIP > 1.0 was considered as differential metabolites for group discrimination. The pathway analysis was performed using MetaboAnalyst for the identified important metabolites using *Arabidopsis thaliana* pathway libraries.

## Additional files

Additional file 1: **Figure S1.** Performance of drought-tolerant (DT) and drought-susceptible (DS) sesame genotypes under drought stress. **Figure S2.** An overview of gene expression in DT and DS. **Figure S3.** Venn diagrams of drought-responsive genes between different time points and genotypes. **Figure S4.** Validation of expression patterns of selected differentially expressed genes using qRT-PCR. **Figure S5.** GO enrichment of up-regulated (a) and down-regulated (b) core genes in response to drought stress in sesame. **Figure S6.** KEGG enrichment of up-regulated (a) and down-regulated (b) core genes in response to drought stress in sesame. **Figure S7.** GO enrichment of up- or down-regulated genes in response to drought stress in DT and DS. **Figure S8.** Venn diagrams of differentially expressed genes between drought-tolerant (DT) and drought-susceptible (DS) genotypes. **Figure S9.** GO and KEGG enrichment of differential expression genes between two genotypes under normal (a and b) and drought stress (c and d) conditions. **Figure S10.** An overview of unique named metabolites detected in DT and DS. **Figure S11.** Partial least square discriminant analysis (PLS-DA) of metabolic profiles in DT and DS in response to drought stress. **Figure S12.** Partial least square discriminant analysis (PLS-DA) of differential metabolites between DT and DS. **Figure S13.** GO enrichment of core drought-responsive gene sets in sesame leaf and root. (DOC 6104 kb)

Additional file 2: **Table S1.** Statistical analyses and mapping results of RNA sequencing reads. (XLSX 12 kb)

Additional file 3: **Table S2.** List of the core gene set of sesame involved in response to drought stress. (XLSX 278 kb)

Additional file 4: **Table S3.** Robust differentially expressed genes between DT and DS during the drought stress. (XLSX 51 kb)

Additional file 5: **Table S4.** Important drought-responsive metabolites identified by PLS-DA in DT. (XLSX 35 kb)

Additional file 6: **Table S5.** Important drought-responsive metabolites identified by PLS-DA in DS. (XLSX 30 kb)

Additional file 7: **Table S6.** Important differential metabolites between DT and DS identified by PLS-DA. (XLSX 24 kb)

Additional file 8: **Table S7.** Detailed results from the metabolomic pathway analysis. (XLSX 13 kb)

Additional file 9: **Table S8.** List of primers used for quantitative real-time RT-PCR analysis. (XLSX 9 kb)

## Data Availability

All RNA sequencing data from the present study have been submitted to the NCBI sequence read archive (SRA) under accession numbers: SAMN09517829 (https://www.ncbi.nlm.nih.gov/biosample/9517829) and SAMN09517828 (https://www.ncbi.nlm.nih.gov/biosample/9517828).

## Abbreviations

ABA: Abscisic acid
DEG: Differentially expressed gene
DS: Drought-susceptible
DT: Drought-tolerant
FDR: False discovery rate
GABA: 4-Aminobutyric acid
GC/MS: Gas chromatography/mass spectrometry
PLS-DA: Partial least squares-discriminant analysis
ROS: Reactive oxygen species
UPLC/MS: Ultra performance liquid chromatography/mass spectrometry
VIP: Variable importance in projection
