# Supplementary material for: Transcriptomic and metabolomic profiling of drought-tolerant and susceptible sesame genotypes in response to drought stress
Source: BMC Plant Biol. 2019 Jun 20;19:267. doi: 10.1186/s12870-019-1880-1 (PMC6585049; doi:10.1186/s12870-019-1880-1)
Supplement: Supplementary file 1 — Figure S1. Performance of drought-tolerant (DT) and drought-susceptible (DS) sesame genotypes under drought stress. Figure S2. An overview of gene expression in DT and DS. Figure S3. Venn diagrams of drought-responsive genes between different time points and genotypes. Figure S4. Validation of expression patterns of selected differentially expressed genes using qRT-PCR. Figure S5. GO enrichment of up-regulated (a) and down-regulated (b) core genes in response to drought stress in sesame. Figure S6. KEGG enrichment of up-regulated (a) and down-regulated (b) core genes in response to drought stress in sesame. Figure S7. GO enrichment of up- or down-regulated genes in response to drought stress in DT and DS. Figure S8. Venn diagrams of differentially expressed genes between drought-tolerant (DT) and drought-susceptible (DS) genotypes. Figure S9. GO and KEGG enrichment of differential expression genes between two genotypes under normal (a and b) and drought stress (c and d) conditions. Figure S10. An overview of unique named metabolites detected in DT and DS. Figure S11. Partial least square discriminant analysis (PLS-DA) of metabolic profiles in DT and DS in response to drought stress. Figure S12. Partial least square discriminant analysis (PLS-DA) of differential metabolites between DT and DS. Figure S13. GO enrichment of core drought-responsive gene sets in sesame leaf and root. (DOC 6104 kb) [file 12870_2019_1880_MOESM1_ESM.doc]

Transcriptomic and metabolomic profiling of drought-tolerant and susceptible sesame genotypes in response to drought stress

Jun You^1^, Yujuan Zhang^1, 2^, Aili Liu^1^, Donghua Li^1^, Xiao Wang^3^, Komivi Dossa^1, 4^, Rong Zhou^1^, Jingyin Yu^1^, Yanxin Zhang^1^, Linhai Wang^1^, Xiurong Zhang^1^*

1. Key Laboratory of Biology and Genetic Improvement of Oil Crops, Ministry of Agriculture and Rural Affairs, Oil Crops Research Institute, Chinese Academy of Agricultural Sciences, Wuhan, 430062, China

2. Special Economic Crop Research Center of Shandon Academy of Agricultural Sciences，Shandong Cotton Research Center, Jinan, 250100, China

3. Quality Inspection and Test Center for Oilseed Products, Ministry of Agriculture and Rural Affairs, Wuhan, 430062, China

4. Centre d’Etudes Régional pour l’Amélioration de l’Adaptation à la Sécheresse (CERAAS), BP 3320 Route de Khombole, Thiès, Sénégal


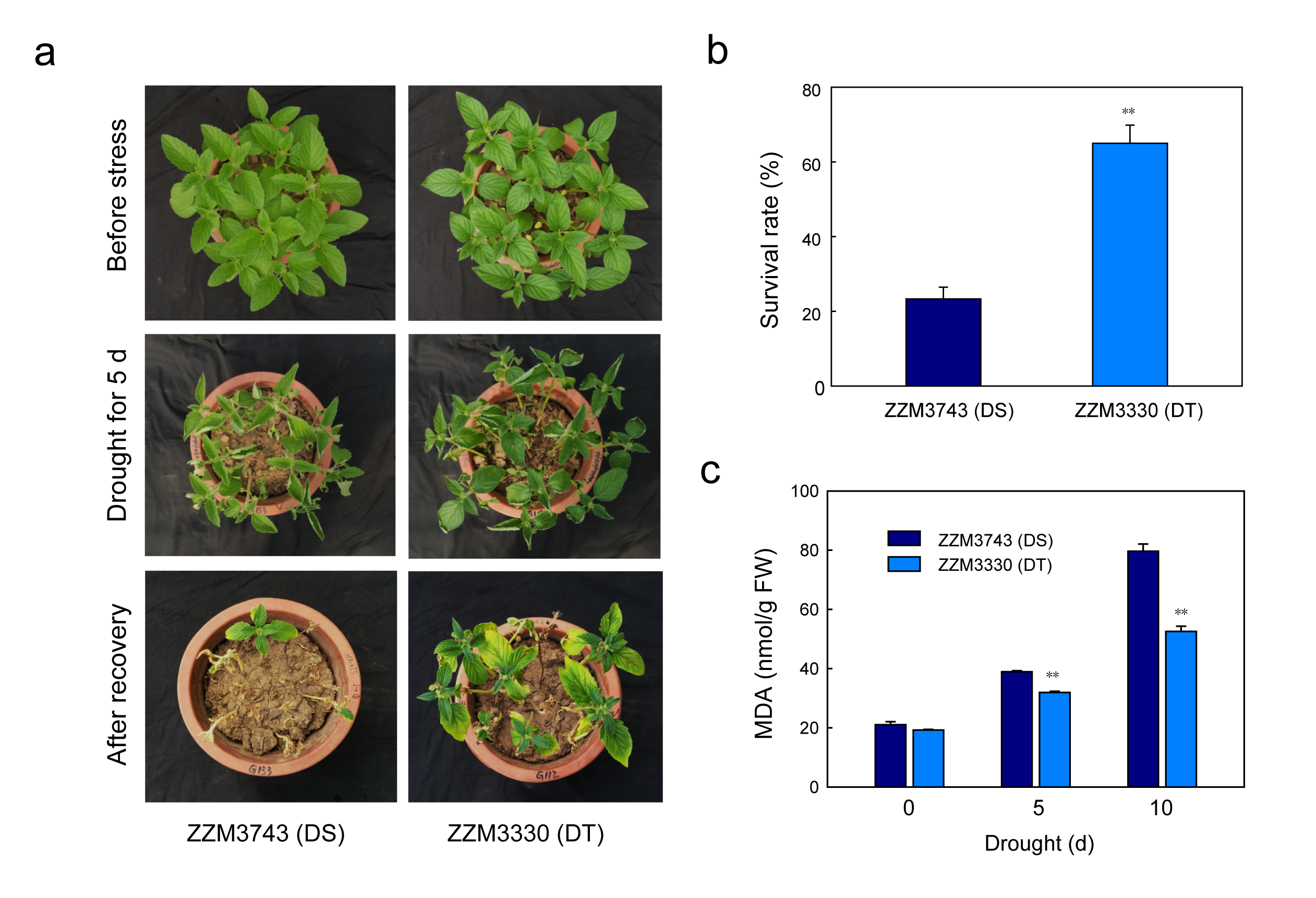


**Figure S1.** Performance of sensitive (DS) and tolerant (DT) sesame genotypes under drought stress. **a** Phenotypes of ZZM3743 (DS) and ZZM3330 (DT) plant under drought stress and after recovery. **b** Survival rate of DS and DT plants after drought stress (*n* = 5). **c** MDA content of DS and DT plants during drought stress treatment (*n* = 3). Data represent mean ± SE. **P* < 0.05; ***P* < 0.01, *t* test.


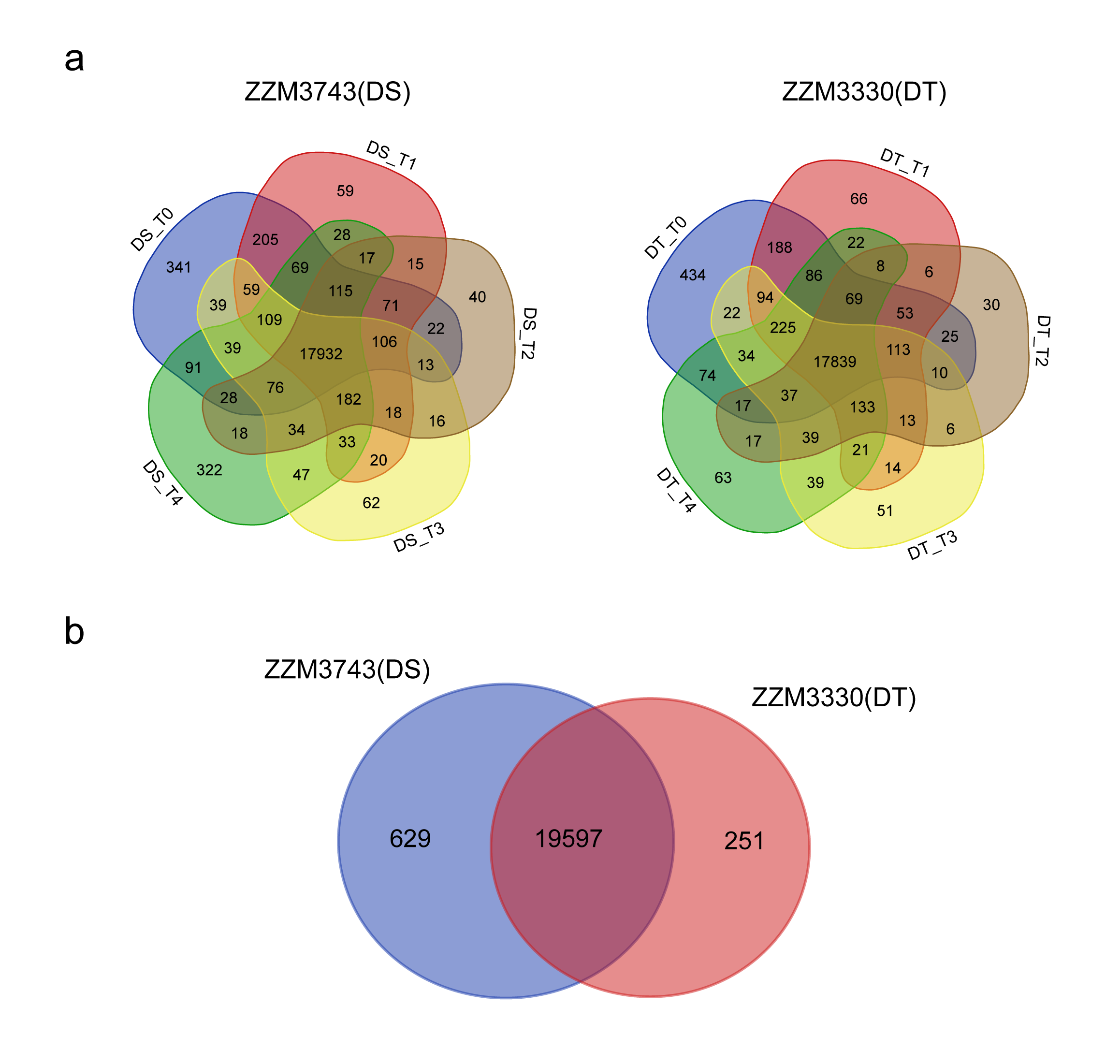


**Figure S2**. An overview of gene expression in DT and DS. **a** The shared and uniquely expressed gene (FPKM > 0.1) numbers during a time-point assay of drought stress in DS and DT, respectively. **b** The shared and uniquely expressed gene (FPKM > 0.1) numbers between two sesame genotypes. DT: drought-tolerant sesame genotype; DS: drought-sensitive sesame genotype

**
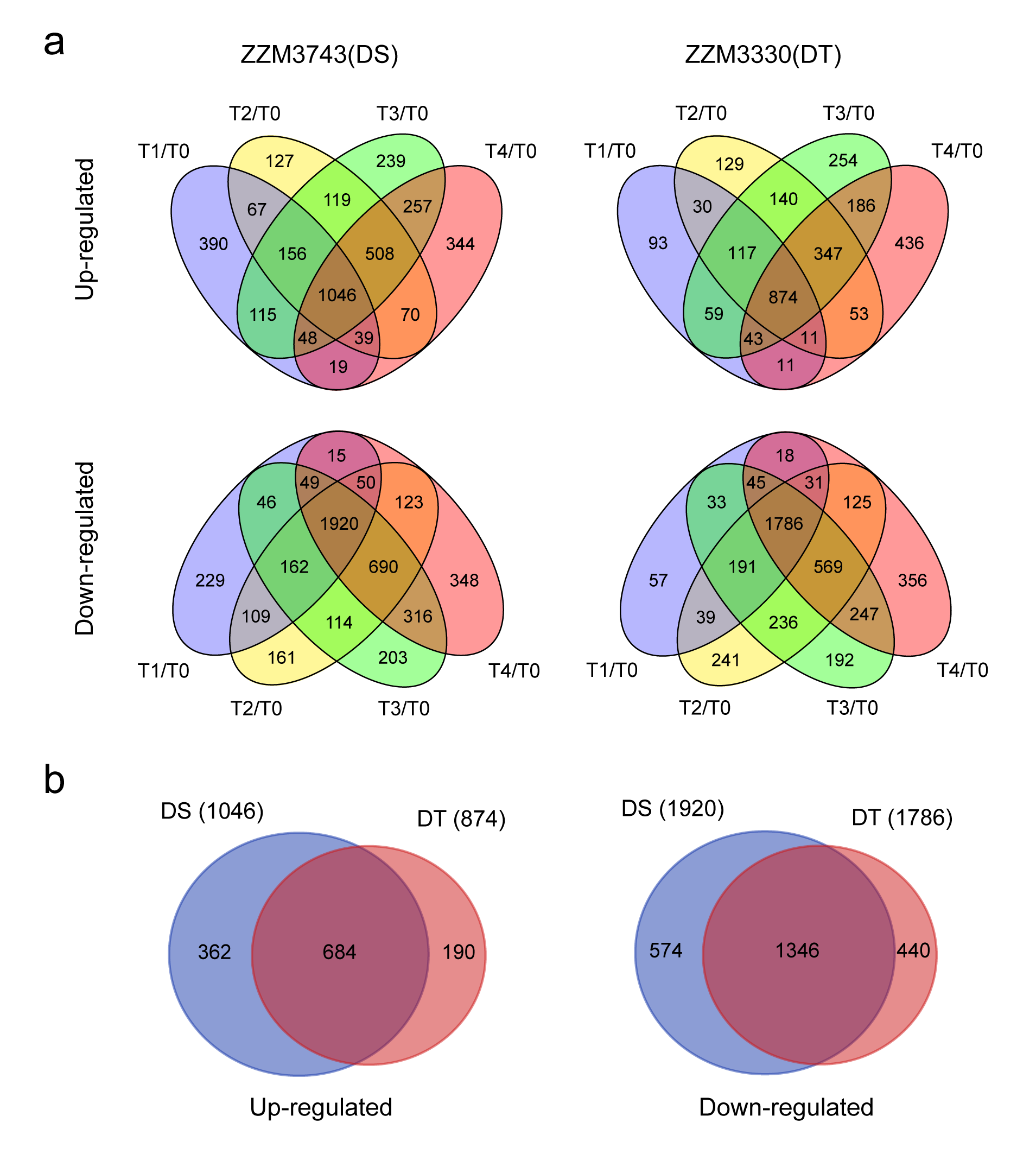
**

**Figure S3.** Venn diagrams of drought-responsive genes between different time points and genotypes. **a** Overlap of up- or down-regulated genes under drought stress among four comparisons (T1/T0, T2/T0, T3/T0 and T4/T0) in DS and DT, respectively. **b** Overlap of up- or down-regulated genes under drought stress during all time-points between DT and DS.


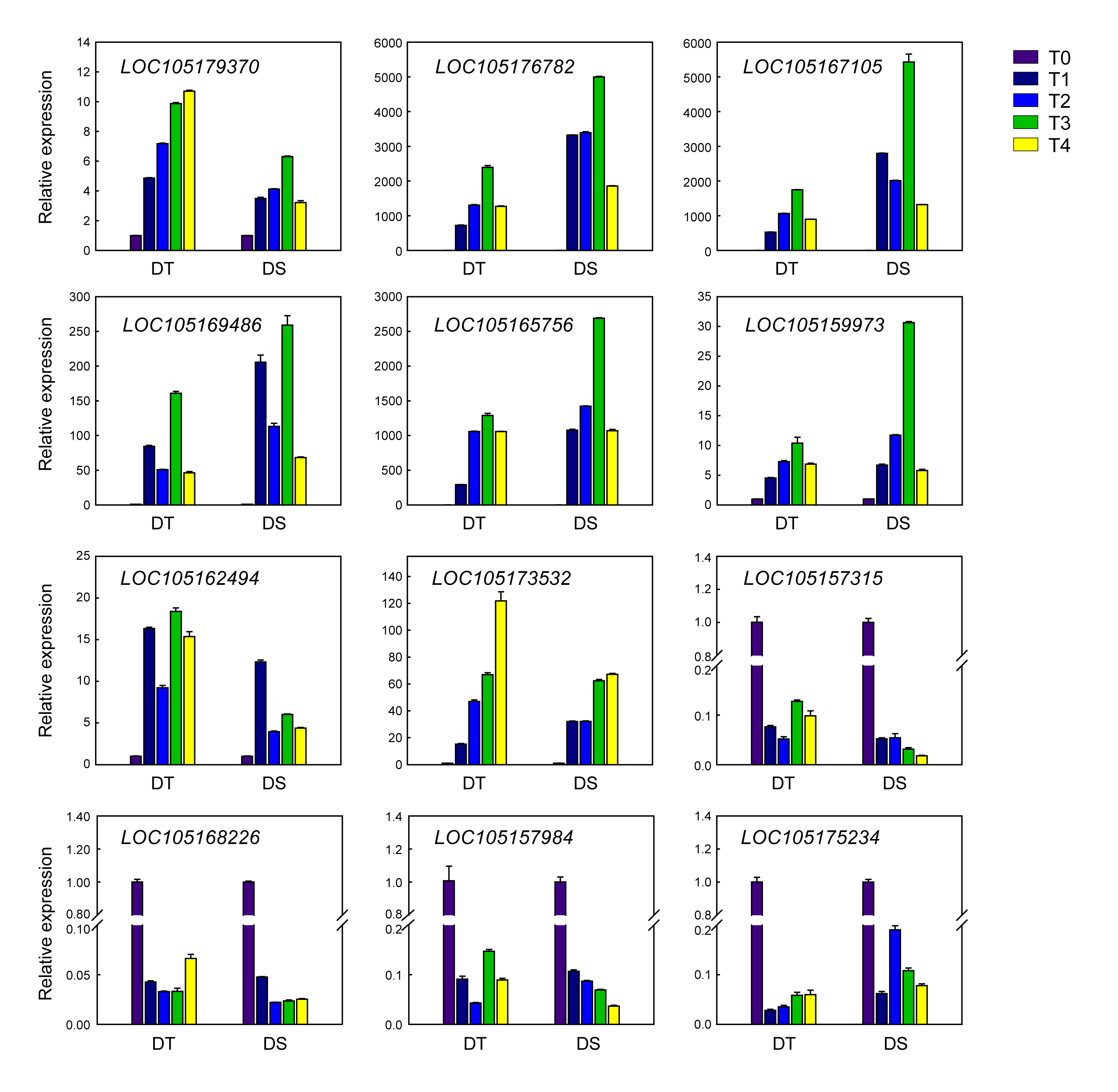


**Figure S4.** Validation of expression patterns of selected differentially expressed genes using qRT-PCR. The expression levels are normalized to sesame *SiH3.3* gene. Error bars indicate SE based on three replicates.


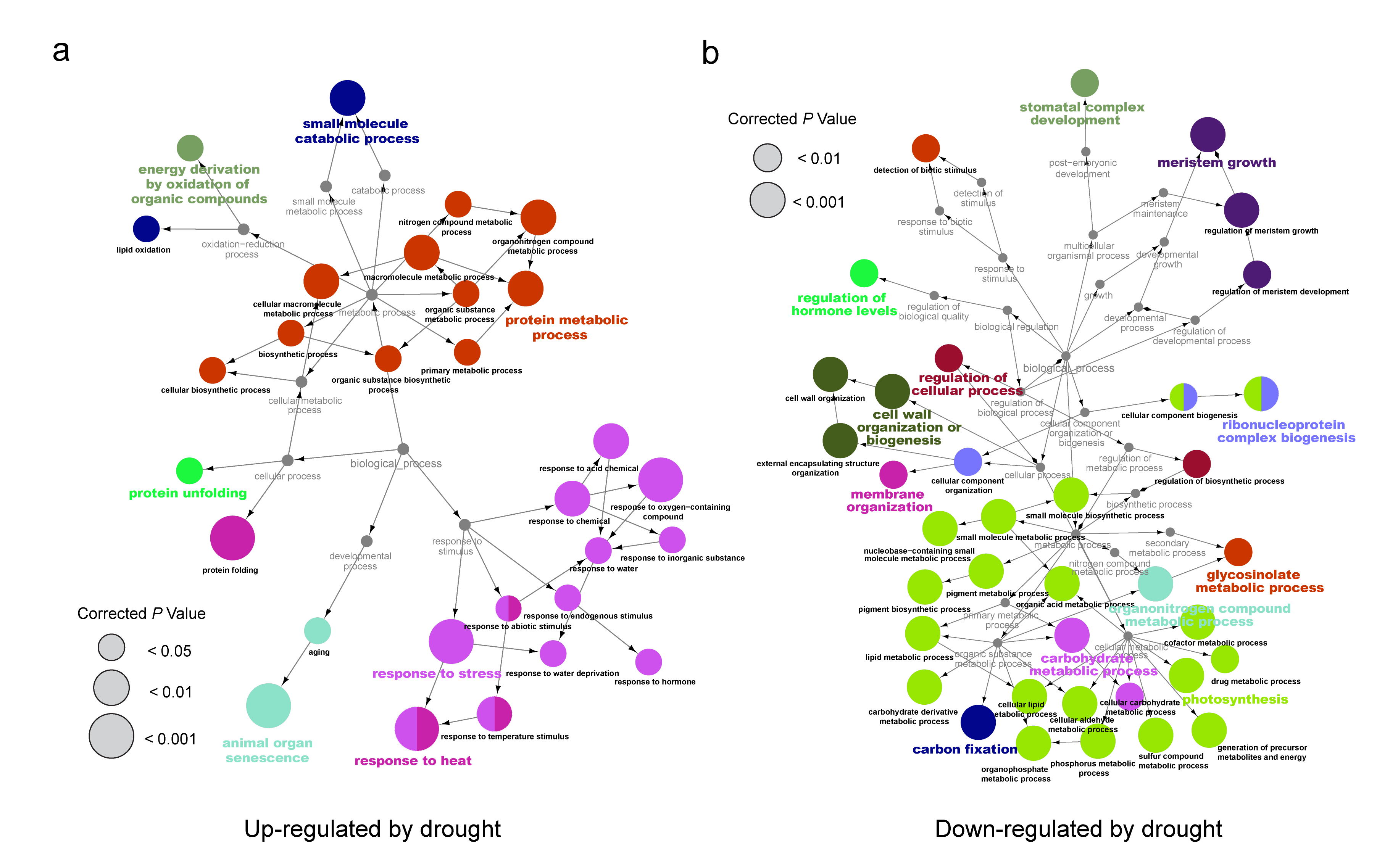


**Figure S5.** GO enrichment of up- regulated (**a**) and down-regulated (**b**) core genes in response to drought stress in sesame. ClueGO analysis generated functionally grouped network with GO terms as nodes linked based on their kappa score level (≥0.3). Colors reflect the label of the most significant term per group. The node size represents the term enrichment significance (Benjamini-Hochberg corrected *p* value based on two-sided hypergeometric tests).

**
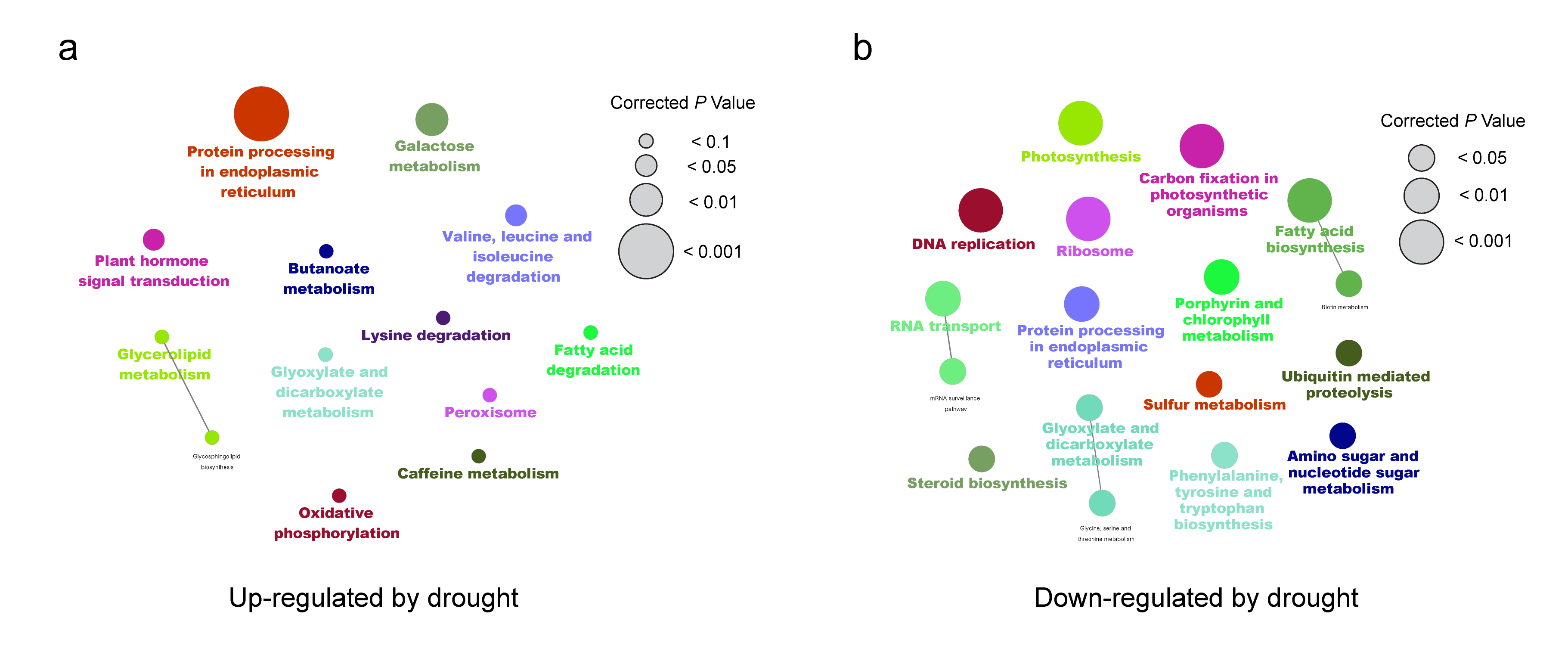
**

**Figure S6.** KEGG enrichment of up- regulated (**a**) and down-regulated (**b**) core genes in response to drought stress in sesame. ClueGO analysis generated functionally grouped network with KEGG terms as nodes linked based on their kappa score level (≥0.3). Colors reflect the label of the most significant term per group. The node size represents the term enrichment significance (Benjamini-Hochberg corrected *p* value based on two-sided hypergeometric tests).


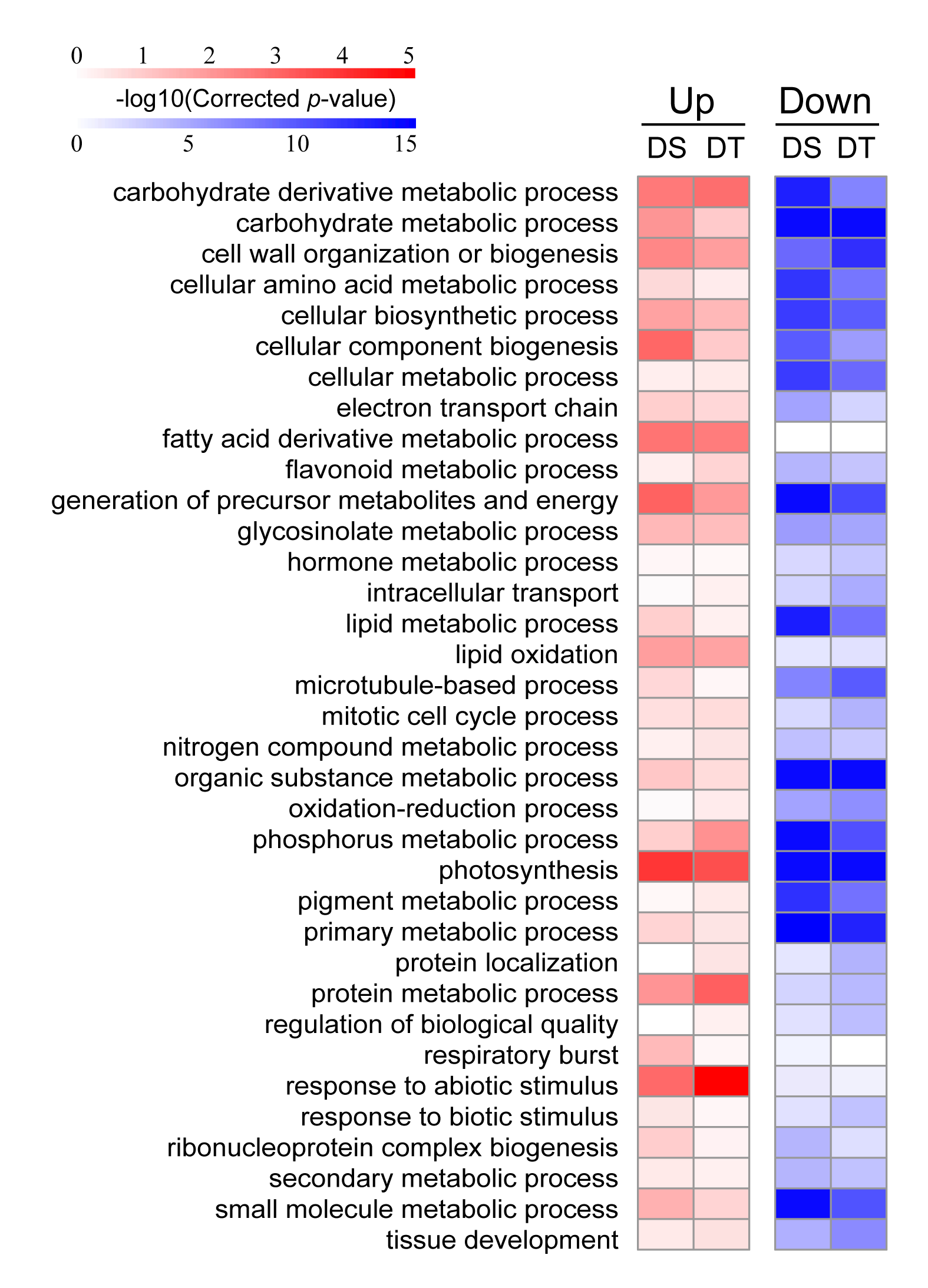


**Figure S7.** GO enrichment of up- or down-regulated genes in response to drought stress in DT and DS. The heatmap presents statistical significance (log10-transformed corrected *p* value) of GO terms over-representation.


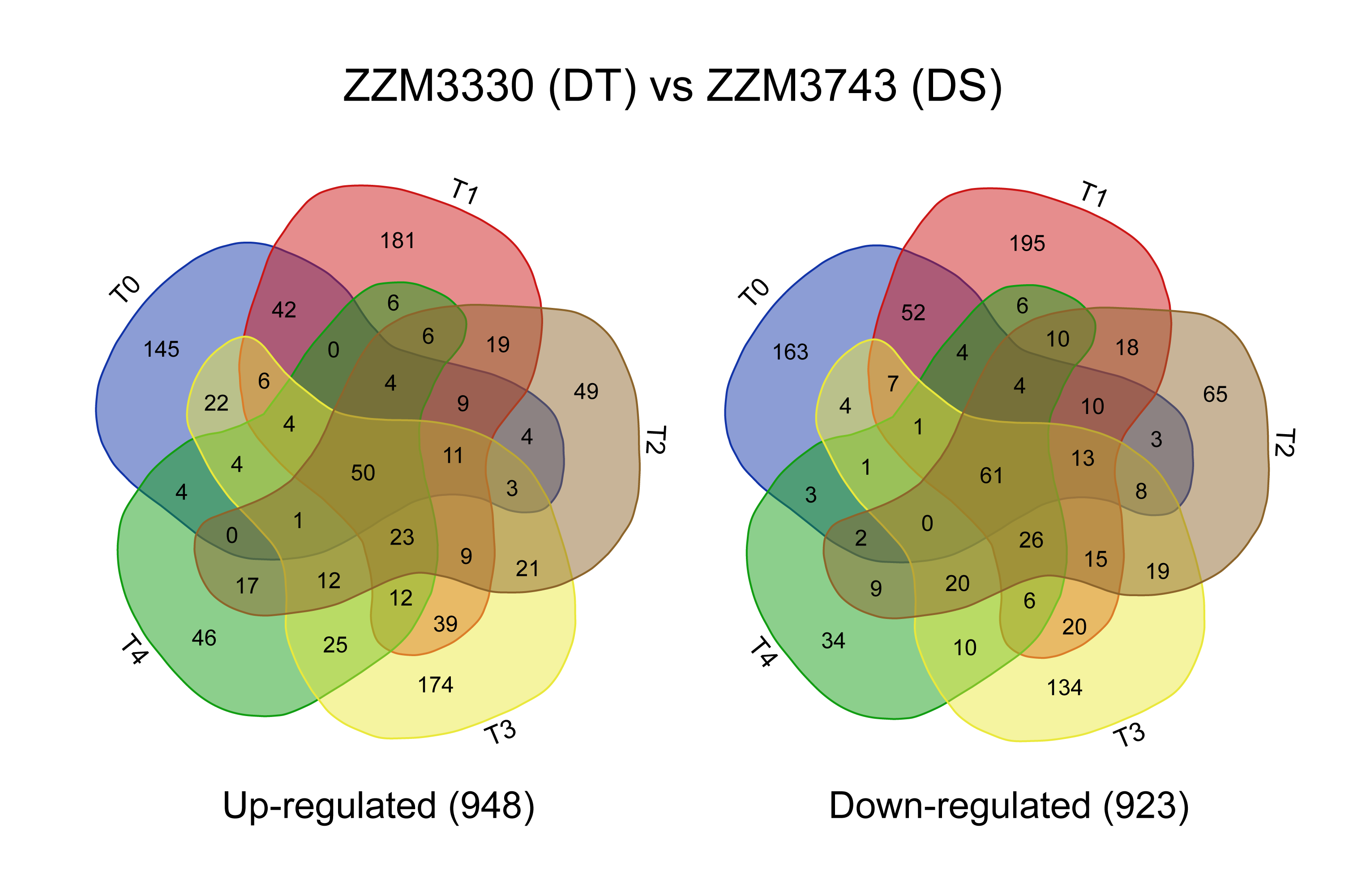


**Figure S8.** Venn diagrams of differentially expressed genes between drought tolerant (DT) and sensitive (DS) genotypes**.**

**
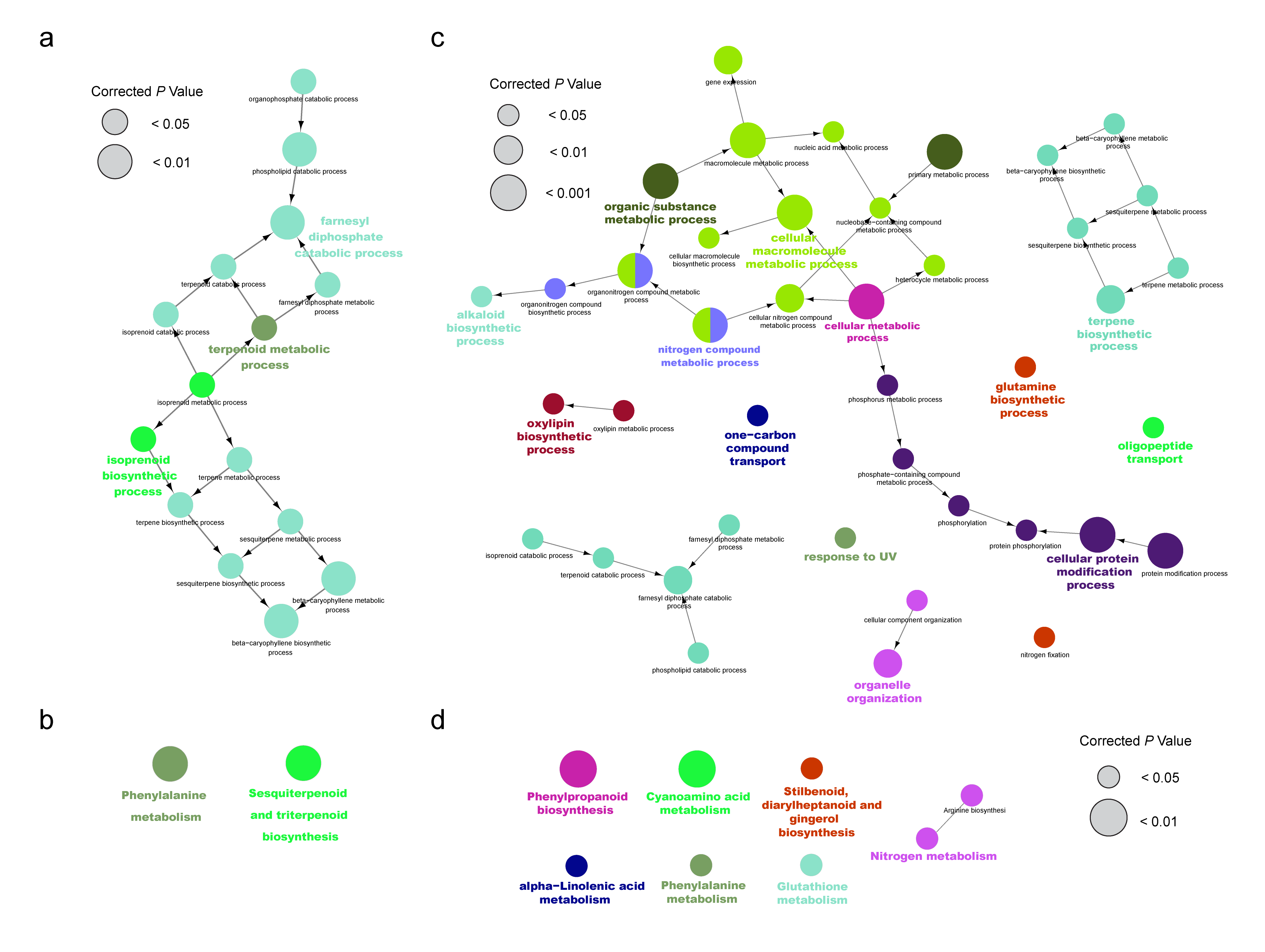
**

**Figure S9.** GO and KEGG enrichment of differential expression genes between two genotypes under normal (**a** and **b**) and drought stress (**c** and **d**) condiitons. ClueGO analysis generated functionally grouped network with GO (**a** and **c**) and KEGG (**b** and **d**) terms as nodes linked based on their kappa score level (≥0.3). Colors reflect the label of the most significant term per group. The node size represents the term enrichment significance (Benjamini-Hochberg corrected *p* value based on two-sided hypergeometric tests).


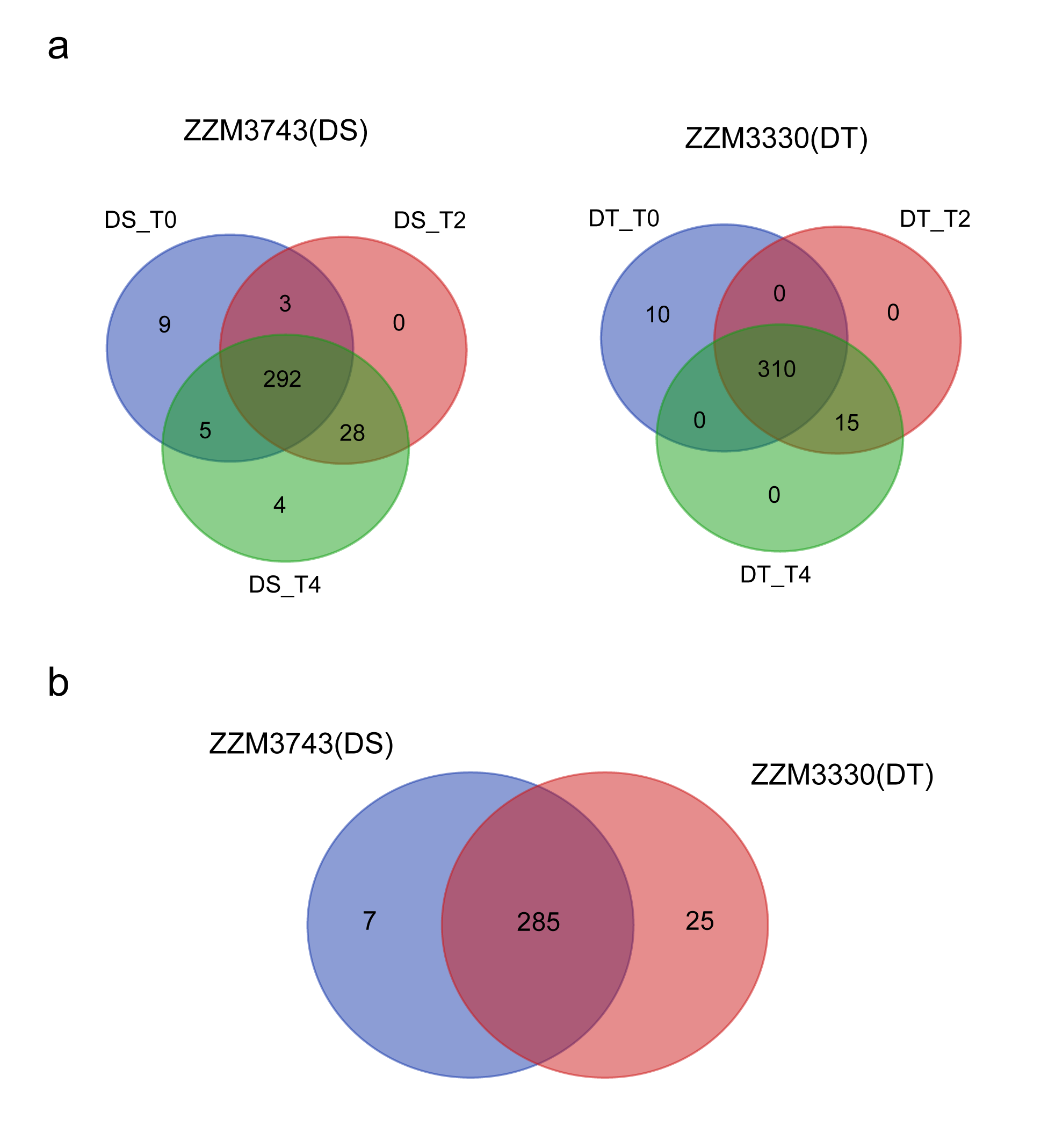


**Figure S10.** An overview of unique named metabolites detected in DT and DS. **a** The shared and uniquely metabolite numbers during a time-point assay of drought stress in DS and DT, respectively. **b** The shared and uniquely metabolite numbers between two sesame genotypes. DT: drought-tolerant sesame genotype; DS: drought-sensitive sesame genotype.


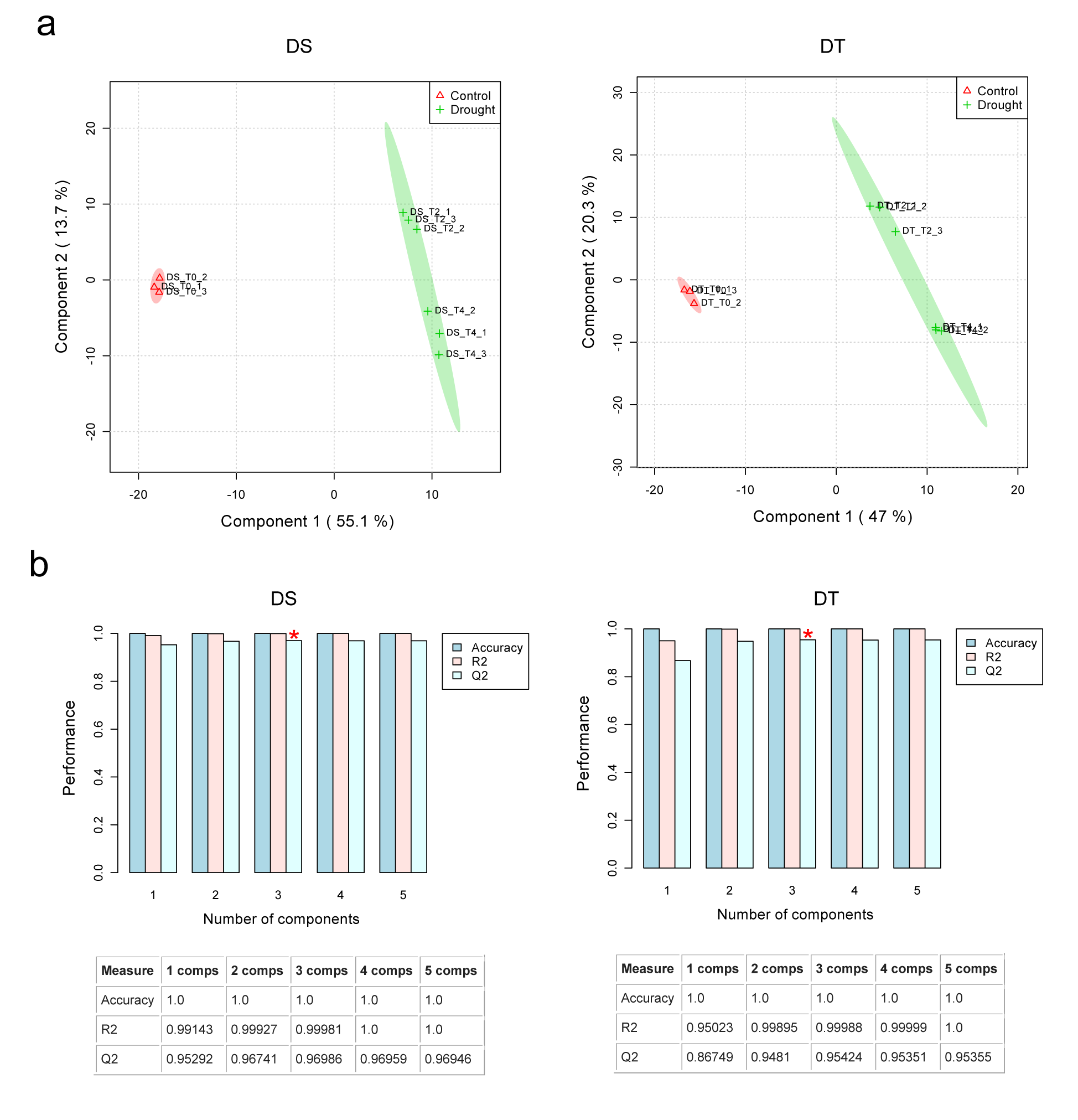


**Figure S11.** Partial least square discriminant analysis (PLS-DA) of metabolic profiles in DT and DS in response to drought stress. **a** PLS-DA of metabolic profiles of DT and DS under control and drought stress at two time points (T2 and T4). b PLS-DA performance measurements. Accuracy, multiple correlation coefficient R2 and the explained variance in prediction Q2 are shown. The red asterisk indicates the best value of selected measure (Q2).

**
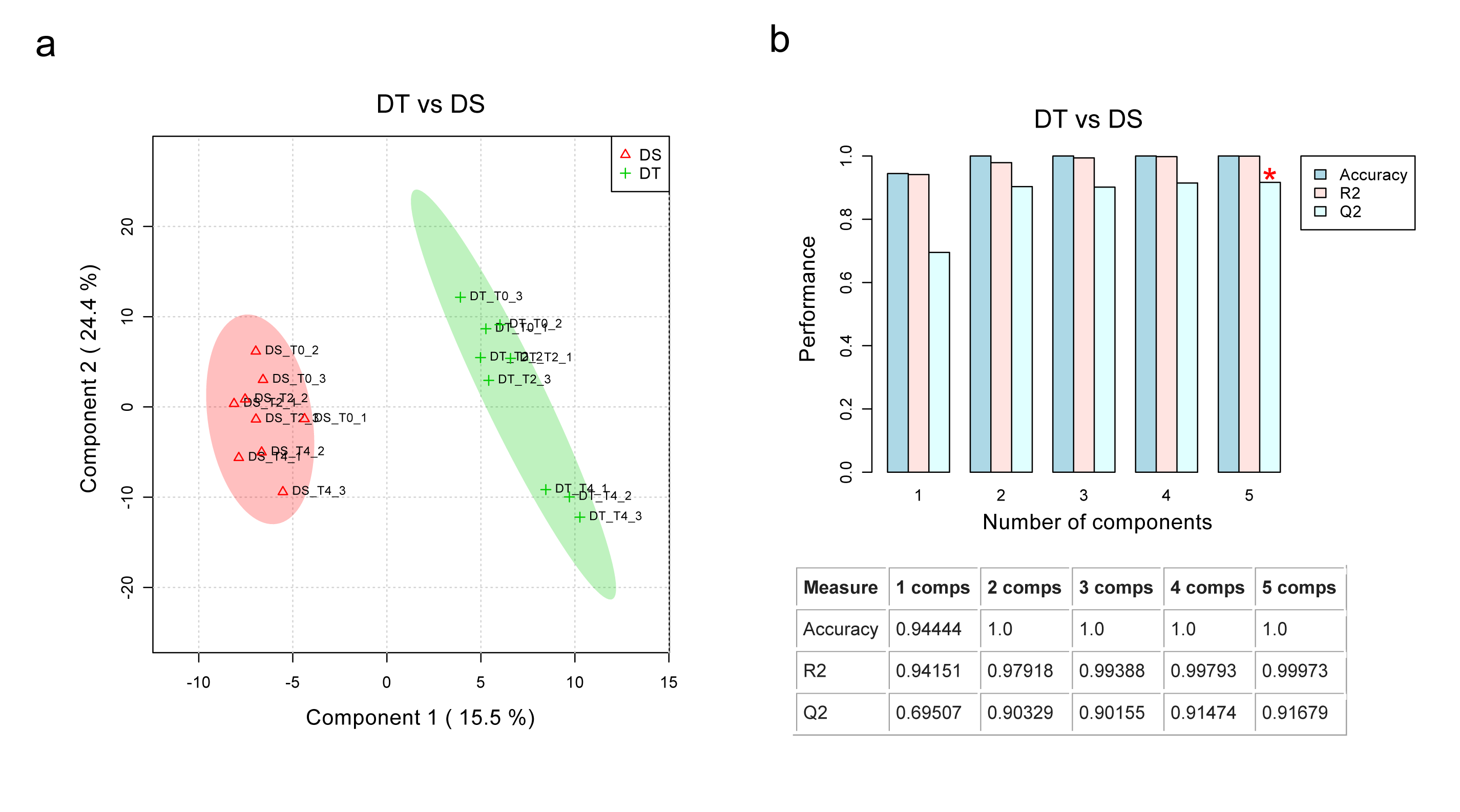
**

**Figure S12.** Partial least square discriminant analysis (PLS-DA) of differential metabolites between DT and DS. **a** PLS-DA and 2D scores loading plot for metabolic profiles of DT and DS. **b** PLS-DA performance measurements. Accuracy, multiple correlation coefficient R2 and the explained variance in prediction Q2 are shown. The red asterisk indicates the best value of selected measure (Q2).

**
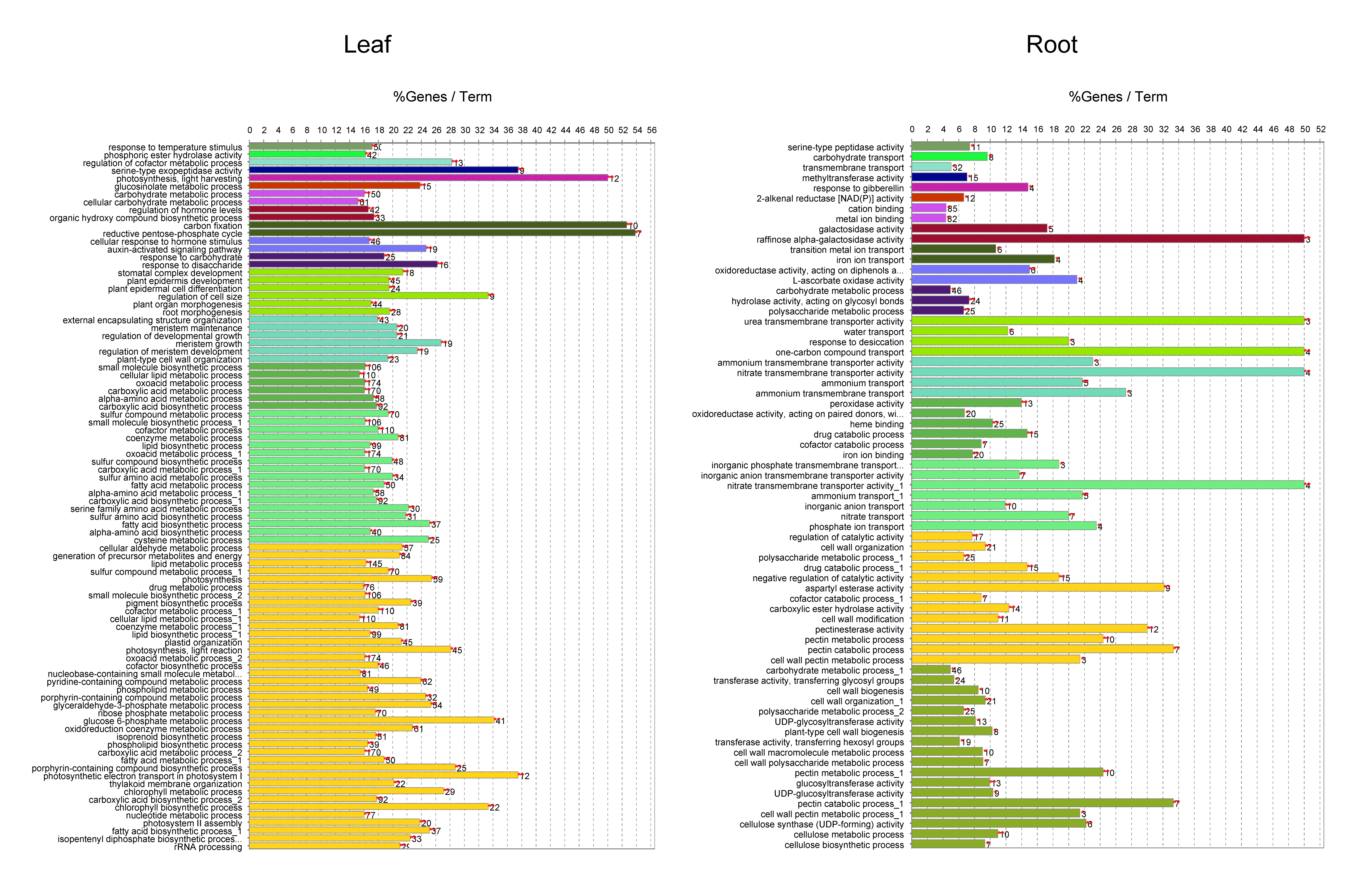
**

**Figure S13.** GO enrichment of core drought-responsive gene set in sesame leaf and root. The bars represent the percentage of genes per term. The number of genes associated with the terms is shown as bar label.
